# A genome-wide association study reveals additive and recessive alleles affecting male fertility in pigs

**DOI:** 10.1186/s40104-025-01312-8

**Published:** 2025-12-15

**Authors:** Pedro Sá, Marta Gòdia, Rodrigo M. Godinho, Claudia A. Sevillano, Barbara Harlizius, Ole Madsen, Henk Bovenhuis

**Affiliations:** 1 Animal Breeding and Genomics, Wageningen University and Research, Wageningen, 6700 AH The Netherlands; 2 Topigs Norsvin Research Center B.V., ‘s-Hertogenbosch, 5216 TZ The Netherlands

**Keywords:** GWAS, Male fertility, *MEIOB*, Pig, Semen traits, *UBE2B*

## Abstract

**Background:**

Understanding the genetic basis of male reproduction in mammals remains challenging. Commercial pig populations offer a unique model for studying fertility, as semen traits are routinely recorded using high-throughput systems.

**Results:**

In a large-scale GWAS of 15 semen traits based on 286,314 ejaculates collected from 2,954 boars of a purebred pig line, we identified 10 QTL, including four loci with recessive deleterious alleles. Several lead SNPs affected multiple semen traits. For example, a SNP on SSC6 was significantly associated with distal cytoplasmic droplets and with effects on tail abnormalities and sperm motility in a follow up analysis. The allele frequencies of some loci were different in older boar’s, most likely due to culling based on poor semen quality. Using WGS, we identified six missense variants in high linkage disequilibrium (LD) with lead SNPs in genes related to sperm production (e.g., *MEIOB*,* CFAP74* and *UBE2B*). Remarkably, the frequency of some alleles with predicted deleterious effects on semen traits increased between 2013 and 2019.

**Conclusions:**

Our results highlight loci with major effects on semen quality, some of which are linked to functional variants in key genes involved in spermatogenesis. The information from this study can be used to select against deleterious alleles affecting semen characteristics in pigs and provides valuable insight into the genetics of mammalian male fertility.

**Supplementary Information:**

The online version contains supplementary material available at 10.1186/s40104-025-01312-8.

## Background

In mammalian species, male fertility plays a critical role in determining reproductive success and overall fitness [1, 2]. In humans, 30% to 50% of infertile males are classified as idiopathic, meaning the underlying cause of subfertility remains to be understood [3]. Low fertility in males have also been documented across a wide range of wild and domestic mammals, with significant consequences in endangered species [4, 5] and local livestock breeds [6] with limited genetic diversity and small effective population sizes. These constraints hinder the discovery of deleterious alleles affecting male reproduction and limit effective conservation strategies.

Commercial pig populations offer a valuable model for investigating the genetic basis of male fertility. In pig breeding, semen quality is key for reproductive success, as it directly impacts fertility results and the transmission of genetic gain. In a commercial setting, pig populations are selected with a strong emphasis on either production or female reproductive traits [7, 8]. Whilst in most breeding schemes there is no selection for male reproduction, boars undergo continuous and routine evaluation of their semen quality [9].

In artificial insemination (AI) centres, semen quality is assessed using automated phenotyping technologies such as computer-assisted sperm analysis (CASA) systems [10]. These systems provide objective and reproducible measurements for many semen parameters on multiple ejaculates from each boar [11].

Over the past decade, the advancement of cost-effective and customized genotyping technologies has led breeding companies to routinely genotype their populations, resulting in the development of extensive genotype datasets [7, 12]. The combination of high-throughput phenotyping and genotyping information offers unique opportunities to study the genetic basis of semen traits. Genome-wide association studies are a well-stablished and proven method for identifying genomic regions affecting specific traits [13]. These studies not only provide valuable biological insights into male reproduction but when combined with whole-genome sequence (WGS) data, can facilitate the discovery of causal variants, including potential deleterious alleles. Endangered wild species and local livestock populations with small population sizes and limited genetic data can benefit from these studies through comparative mapping [14, 15].

In this study, we identified quantitative trait loci (QTL) and described their effects on semen traits in a purebred pig line. It has been reported that the additive genetic effect of QTL affecting boar semen traits can change with age of the boar [16, 17]. Therefore, we also addressed whether the effect of identified loci changed with the age of the boar. Additionally, we explored the changes in allele frequencies of lead SNPs over time and provided a functional analyses of these variants [18], highlighting potential causal variants from WGS data affecting semen traits in pigs.

## Methods

### Genotypes and phenotypes

Genotypic and phenotypic information on boars from AI centres was provided by Topigs Norsvin. A total of 3,010 boars from a Large White-based line were genotyped using either Illumina GeneSeek custom 50 K or 25 K SNP chip (Lincoln, NE, USA). These chips yielded genotypes for 50,689 SNPs and 26,894 SNPs, respectively, mapped to the current reference genome (Sscrofa11.1). Quality control using PLINK v.1.9 [19] consisted of excluding SNPs located on sex chromosomes or unmapped SNPs, SNPs with a minor allele frequency (MAF) lower than 0.01 and a call rate lower than 0.9. Subsequently, these genotypes were imputed to 660 K SNPs. The imputation was performed in two steps, first to 50 K and then to 660 K, both with FImpute v3.0 [20]. The reference population for imputation consisted of 800 animals from the same purebred line genotyped with the Axiom porcine 660 K SNP array from Affymetrix (Santa Clara, CA, USA). This imputation yielded genotypes on 554,768 SNPs. A total of 67,068 SNPs were removed due to MAF < 0.01 and 39 boars were removed due to mismatch rate above 0.05 between the original and imputed panel. In addition, for boars with both imputed and whole-genome sequencing data (*n* = 104), imputation quality was assessed by genotype concordance between imputed and sequencing SNPs, yielding an overall concordance of 0.985. After quality control and imputation, the final dataset used for the genome-wide association included genotypes on 487,700 SNPs from 2,971 boars.

Fresh ejaculates were collected from these boars between September 2009 and January 2022. These ejaculates were collected as a routine practise using a semiautomatic collection system and immediately pre-diluted 1:1 using Solusem Bio + (IMV). The evaluation of fresh semen was performed using IVOS CASA system on pre-diluted ejaculates (Hamilton Thorne Inc., Beverly, MA, USA). Additionally, head abnormalities were assessed with microscopy by experienced technicians. The process included staining a sub-sample of the ejaculate and identifying and counting sperm cells with abnormal head morphology. After the evaluation of fresh semen, ejaculates were fully diluted to an end dose concentration of 1.3 to 1.5 billion sperm cells and stored in commercial doses of 80 mL at 17 °C. After 3 days of storage, a random subset of these commercial doses were evaluated again using the CASA system.

The dataset consisted of three classes of semen traits, i.e. semen quantity, sperm motility and sperm morphology. The total number of sperm cells, ejaculate volume and concentration were included as semen quantity parameters. Sperm motility traits were total and progressive motility of fresh semen. Sperm morphology traits included the total proportion of morphological abnormalities, total cytoplasmatic droplets, proximal and distal cytoplasmatic droplets, distal midpiece reflex, bent tail, and to complement these CASA traits, the microscopic assessment for abnormal acrosome and abnormal head morphologies. The phenotypic data measured after three days of storage included total and progressive motility after storage traits. The final phenotypic dataset included records from a total of 286,314 ejaculates from 2,954 boars, with an average of 97 ejaculates per boar.

### Genetic parameters and GWAS

Analyses were performed using the animal repeatability model described in Eq. 1:

1 $${\boldsymbol{y}}={\varvec{X}}{\varvec{\beta}}+{\varvec{Z}}{\varvec{a}}+{\varvec{W}}{\varvec{p}}+ {\varvec{e}}$$

where ***y*** represents a vector with phenotypic observations and ***X*** is the incidence matrix for the fixed effects; ***Z*** and ***W*** are incidence matrices for random additive genetic and permanent environment effects, respectively; ${\boldsymbol{\beta}}$, $\boldsymbol{a}$, and $\boldsymbol{p}$ are the vectors with solutions for fixed (${\varvec{\upbeta}}$) and random effects ($\mathbf{a}$ and $\mathbf{p}$). Random additive genetics and permanent environment effects were assumed to be distributed as $\mathbf{a}\sim \text{N}\left(0, \mathbf{G}{\upsigma }_{\text{a}}^{2}\right)$ where **G** is the genomic relationship matrix and ${\upsigma }_{\text{a}}^{2}$ is the additive genetic variance and $\mathbf{p}\sim \text{N}\left(0, \mathbf{I}{\upsigma }_{\text{pe}}^{2}\right)$ where **I** is the identity matrix and ${\upsigma }_{\text{pe}}^{2}$ is the permanent environment variance. To account for population structure, we used the commercial chip data (20,963 SNPs), which provided sufficient marker density for reliable relationship estimation [21]. The **G** matrix was constructed using the calc_grm function of MixBLUP v.2.2 [22] and following the method described by Yang and co-authors [23]. The residual effect was assumed to be distributed as $\mathbf{e}\sim \text{N}\left(0, \mathbf{I}{\upsigma }_{\text{e}}^{2}\right)$, where **I** is the identity matrix and ${\upsigma }_{\text{e}}^{2}$ is the variance of residual effects.

The fixed effects included a covariate of age of the boar at collection, from 7 to 60 months, a class variable for number of days in between collections, between 1 and 15 d, a class variable for the calendar month of collection, 12 months of the year, a class variable for the person responsible for collecting the semen, 173 collectors, a class variable for the lab technician that measured semen quality with CASA and microscopy, 42 lab technicians, and a class variable for the herd-year-season of birth of the boar. The effect of the age of the boar at collection was fitted using a Wilmink function [24]. In a previous study, we observed that this function can appropriately describe the changes in semen traits with age of the boar [25].

Variance components estimation and GWAS were performed using Wombat v03-11-2023 [26]. The estimated variance components were used in the subsequent GWAS also performed using the model in Eq. 1. Semen quality traits were not normally distributed. Cubic transformations were applied on motility traits, and log-transformations were applied on sperm morphology traits. Based on variance components from Eq. 1, we calculated heritabilities (h^2^) and repeatabilities (rep) for semen traits as:

$${\text{h}}^{2}=\frac{{\upsigma }_{\text{a}}^{2}}{{\upsigma }_{\text{p}}^{2}}\ \text{and rep}=\frac{{\upsigma }_{\text{a}}^{2}+ {\upsigma }_{\text{pe}}^{2}}{{\upsigma }_{\text{p}}^{2}},\text{ with},\ {\upsigma }_{\text{p}}^{2}={\upsigma }_{\text{a}}^{2}+{\upsigma }_{\text{pe}}^{2}+ {\upsigma }_{\text{e}}^{2}$$

where ${\upsigma }_{\text{p}}^{2}$ is the total phenotypic variance and ${\upsigma }_{\text{a}}^{2}$, ${\upsigma }_{\text{pe}}^{2}$ and ${\upsigma }_{\text{e}}^{2}$ are the additive genetic, permanent environment and residual variances, respectively.

Subsequently GWAS were performed, where the effect of SNPs on semen traits were tested by adding each SNP as a covariable to the model in Eq. 1. For each SNP, an additive (coded as −1, 0 and 1) and a dominance/recessive effect (coded as −1/2, 1 and −1/2) were fitted. The variance components were fixed to the values estimated previously with the same model.

SNPs with fewer than five animals for any of the genotype classes were removed for the GWAS analysis. The threshold for significant associations was adjusted for multiple testing using Bonferroni which is conservative in the context of GWAS as SNPs are often in LD, but provides stringent control of false positives. Considering a nominal significance threshold of *P*-value < 0.05 and testing of 487,700 independent SNPs, a SNP association was considered significant when *P*-value < 1.0 × 10^−7^ (−log_10_(*P*-value) > 7.0). Additionally, a suggestive threshold was defined based on a false discovery rate (FDR) < 0.05 using the R package RAINBOWR v0.1.29 [27].

### QTL analysis and SNP effects

In this section, we will first describe how we defined QTL regions for significant lead SNPs in order to identify candidate genes. Subsequently, we will describe the genotypic effects and the variance explained by lead SNPs on semen traits.

Significant SNPs were considered to reflect the same causal mutation if the distance between them was less than 2 Mb. Additionally, based on common practice and the LD patterns observed in our data, SNPs were considered to reflect the same QTL if the pairwise LD (*r*^*2*^) was larger than 0.65. The LD analysis was performed using PLINK v.1.9 [19]. The final QTL regions were subsequently expanded by 1 Mb upstream and downstream of the first and last significant SNP within these regions. For each QTL, the first significant SNP refers to the most upstream SNP below the Bonferroni significance threshold, and the last significant SNP refers to the most downstream SNP below the Bonferroni threshold. For constructing regions around suggestive SNPs, the same procedure was applied.

In a follow-up analysis, the genetic variance of lead SNPs combining additive and dominance/recessive effects was estimated by fitting the SNP as a class variable in Eq. 1. The average (${\mu }_{SNP}$) and the variance explained by a lead SNP (${\sigma }_{SNP}^{2}$) were calculated as:

$$\begin{array}{c}{\upmu }_{\text{SNP}}={\text{f}}_{\text{AA}}\cdot {\upbeta }_{\text{AA}}+ {\text{f}}_{\text{Aa}}\cdot {\upbeta }_{\text{Aa}}+ {\text{f}}_{\text{aa}}\cdot {\upbeta }_{\text{aa}}, \text{and}\\ {\upsigma }_{\text{SNP}}^{2}= {\text{f}}_{\text{AA}}\cdot {{(\upbeta }_{\text{AA}}-{\upmu }_{\text{SNP}})}^{2}+ {\text{f}}_{\text{Aa}}\cdot {{(\upbeta }_{\text{Aa}}-{\upmu }_{\text{SNP}})}^{2}+ {\text{f}}_{\text{aa}}\cdot {{(\upbeta }_{\text{aa}}-{\upmu }_{\text{SNP}})}^{2}\end{array}$$

where ${\text{f}}_{\text{AA}}$, ${\text{f}}_{\text{Aa}}$ and ${\text{f}}_{\text{aa}}$ are the genotype frequencies of the homozygous for the major allele AA (coded as 0), the heterozygous Aa (coded as 1) and the homozygous for the minor allele aa (coded as 2) and ${\upbeta }_{\text{AA}}$, ${\upbeta }_{\text{Aa}}$ and ${\upbeta }_{\text{aa}}$ are the estimated effect sizes of these genotypes, respectively. The percentage of phenotypic variance explained by a lead SNP was calculated as:

$${\text{h}}_{\text{SNP}}^{2}=\frac{{\upsigma }_{\text{SNP}}^{2}}{{\upsigma }_{\text{p}}^{2}}\cdot 100\%,\,{\upsigma }_{\text{p}}^{2}={\upsigma }_{\text{a}}^{2}+{\upsigma }_{\text{pe}}^{2}+ {\upsigma }_{\text{e}}^{2}$$

where ${\upsigma }_{\text{p}}^{2}$ is the total phenotypic variance and ${\upsigma }_{\text{a}}^{2}$, ${\upsigma }_{\text{pe}}^{2}$ and ${\upsigma }_{\text{e}}^{2}$ are the additive genetic, permanent environment and residual variances, respectively. For easier interpretation, the effects of genotype classes were estimated on untransformed traits and the percentage of phenotypic variance explained by lead SNPs was estimated on untransformed semen quantity and transformed sperm motility and morphology traits. These analyses were performed in ASREML v.4.2 [28]. Variance components were re-estimated in these models. For computational efficiency, family relationships were modelled based on pedigree rather than genomic information. The pedigree-based relationship matrix was constructed based on a dataset that spanned 26 generations and included 17,701 individuals.

### Genotype frequencies and effect of lead SNPs with age of the boar

In AI centres, boars with poor semen quality are culled and only a selected set of boars are collected at older ages. This practice may affect genotype frequencies of older boars [16]. In addition, genetic variance may also change with age of the boar [16]. In this section, we describe genotype frequencies and genotype effects of lead SNPs at different ages (between 7 and 60 months).

Records of semen traits were divided into three age categories: ejaculates collected from boars between 7 and 13 months of age, between 14 and 23 months and 24 to 60 months of age. The records from these three ages were fitted in a multivariate analysis and the effect of each lead SNP was tested as a class effect. Unlike the analysis of the complete dataset, analyses restricted to a single age category showed collectors to be confounded with lab technicians. Therefore, we included a collector-lab technician term as a random effect in Eq. 1, assuming collector-lab technician effects to be distributed $\text{N}\left(0,\mathbf{I}{\upsigma }_{\text{clt}}^{2}\right)$, where **I** is the identity matrix and ${\upsigma }_{\text{clt}}^{2}$ is the collector-lab technician variance. Moreover, each age category included many herd-year-season classes with fewer observations each, so herd-year-season of birth was also modelled as a random effect in Eq. 1 and assumed to be distributed $\text{N}\left(0,\mathbf{I}{\upsigma }_{\text{hys}}^{2}\right)$, where **I** is the identity matrix and ${\upsigma }_{\text{hys}}^{2}$ is the variance of herd-year-season of birth.

This analysis was performed using ASREML v.4.2 [28]. The covariances of all random effects between ages were fixed to values previously estimated. The effects of genotype classes of lead SNPs per age were estimated based on untransformed semen quantity and sperm morphology traits.

### Genetic trends for QTL

In this population, the breeding goal does not include semen traits. Nevertheless, allele frequencies for lead SNPs may change due to correlated responses or simply due to genetic drift. In this section, we describe how allele frequencies of lead SNPs change with birth year of the boar.

We calculated the allele frequency of the minor allele for each lead SNP as a function of year of birth of the boars. In this population, boars were born between 2005 and 2020. Genotyping of boars became a standard practice in 2015 and genotyped boars born before 2013 represented a selected set of boars with consistently good semen quality. Due to this bias, genotyped boars born from 2005 to 2012 were removed. The allele frequency in each year was calculated if at least 100 boars were born in that year. Genotyped boars born in 2020 were removed because of this criterion. Finally, the frequency of the minor allele per year of birth was calculated between 2013 and 2019 using PLINK v1.9 [19]. The standard error of the minor allele frequency was calculated as:

$${\text{SE}}_{f_a}=\sqrt{\frac{f_a(1-f_a)}{2\boldsymbol N}}$$

where ${{\varvec{f}}}_{{\varvec{a}}}$ is the allele frequency of the minor allele and ***N*** is the total number of boars born in that year.

### Effect of lead SNPs on other semen traits

To understand if lead SNPs affected multiple semen traits, we compared the −log_10_(*P*-value) of lead SNPs across all semen traits included in this study. We considered that a lead SNP had an effect on semen traits other than the one in its initial discovery when the *P*-value < 1.0 × 10^−3^ (−log_10_(*P*-value) > 3.0). Additionally, the genotypic effects were estimated following the model in Eq.1 as described previously.

### Functional analysis

While GWAS using high-density SNP data is a proven method for detecting QTL affecting traits, they are unlikely to reveal the true causal variant [13]. In order to identify candidate causal variants and affected genes, we performed a functional analysis using additional WGS data. Topigs Norsvin provided WGS data from 307 boars belonging to the same line. The dataset included 24,239,067 SNPs. We used the pig-specific Combined Annotation Dependent Depletion (pCADD) pipeline [18] with default parameters to identify and score WGS variants. Briefly, we considered variants that were in high LD with lead SNPs, i.e., *r*^*2*^ > 0.70, and within 2 Mb upstream and downstream of the lead SNP. The pCADD pipeline is a method for prioritising variants based on how likely these are to be functional, i.e., have an effect on an animal’s fitness. The pCADD scores are a combination of several metrics, including conservation scores and functional impacts on protein sequences, and range from 0 to approximately 95, with high scores indicating variants more likely to be deleterious or functional. For context, pCADD scores of 20 indicate variants in the top 1% most impactful [18]. The pCADD also considers Sorting Intolerance From Tolerance (SIFT) scores [29]. For comparison, we also reported the SIFT scores for WGS variants in high LD.

## Results

Descriptive statistics of semen records, heritability and repeatability estimates are in Table 1. Heritability estimates ranged from 0.04 to 0.28 and repeatability estimates ranged from 0.15 to 0.64. Semen quantity and sperm motility traits were moderately heritable (0.12 or 0.13 for semen quantity traits; from 0.15 to 0.17 for sperm motility traits). Heritability estimates for sperm morphology traits varied considerably between traits. Heritability estimates were highest for distal midpiece reflex and proximal cytoplasmic droplets (0.28 and 0.20, respectively) and lowest for bent tail and abnormal acrosome (0.08 and 0.04, respectively).

**Table 1:** Description of semen records of the boars in the Large White-based line, estimates of heritability (h^2^) and repeatability (rep)

Trait^a^	**Number of boars**	**Number of observation**	**Mean**	**SD**^**b**^	**h**^**2 c**^	**rep **^**c**^
Semen quantity						
Volume, mL	2,936	285,921	401.8	119.2	0.12 _(0.01)_	0.35 _(0.02)_
Concentration, ×10^6^/mL	2,936	285,891	178.2	67.5	0.13 _(0.01)_	0.36 _(0.02)_
Number of sperm cells, ×10^9^	2,936	286,025	68.9	25.4	0.13 _(0.01)_	0.37 _(0.02)_
Sperm motility, %						
Total motility of fresh semen	2,921	270,826	90.6	4.6	0.15 _(0.02)_	0.58 _(0.02)_
Total motility after 3 days of storage	2,905	135,352	81.4	9.8	0.15 _(0.01)_	0.38 _(0.02)_
Progressive motility of fresh semen	2,935	283,453	81.6	8.0	0.17 _(0.02)_	0.49 _(0.02)_
Progressive motility after 3 days of storage	2,905	135,468	72.4	10.6	0.17 _(0.01)_	0.40 _(0.02)_
Sperm morphology, %						
Total morphological abnormalities	2,865	103,420	15.8	10.9	0.16 _(0.02)_	0.50 _(0.02)_
Total cytoplasmatic droplets	1,887	148,777	8.5	5.3	0.18 _(0.02)_	0.58 _(0.03)_
Proximal cytoplasmatic droplets	1,886	148,245	4.2	3.1	0.20 _(0.02)_	0.58 _(0.03)_
Distal cytoplasmatic droplets	1,886	149,114	4.1	2.8	0.19 _(0.02)_	0.51 _(0.03)_
Distal midpiece reflex	1,885	147,918	2.9	2.6	0.28 _(0.03)_	0.64 _(0.04)_
Bent tail	1,885	137,028	0.8	0.6	0.08 _(0.01)_	0.27 _(0.02)_
Abnormal head	2,851	73,095	1.1	1.7	0.11 _(0.01)_	0.24 _(0.01)_
Abnormal acrosome	715	3,231	1.4	0.9	0.04 _(0.03)_	0.15 _(0.04)_

^a^All semen quality traits were measured with CASA systems, except abnormal head and abnormal acrosome which were measured using standard microscopy assessments. Semen traits of fresh semen were measured after pre-dilution

^b^*SD* Standard deviation of the trait

^c^Genetic parameters were estimated for untransformed semen quantity and transformed sperm motility and sperm morphology traits. Standard errors are shown in subscript

### GWAS and SNP effects

**Fig. 1:**
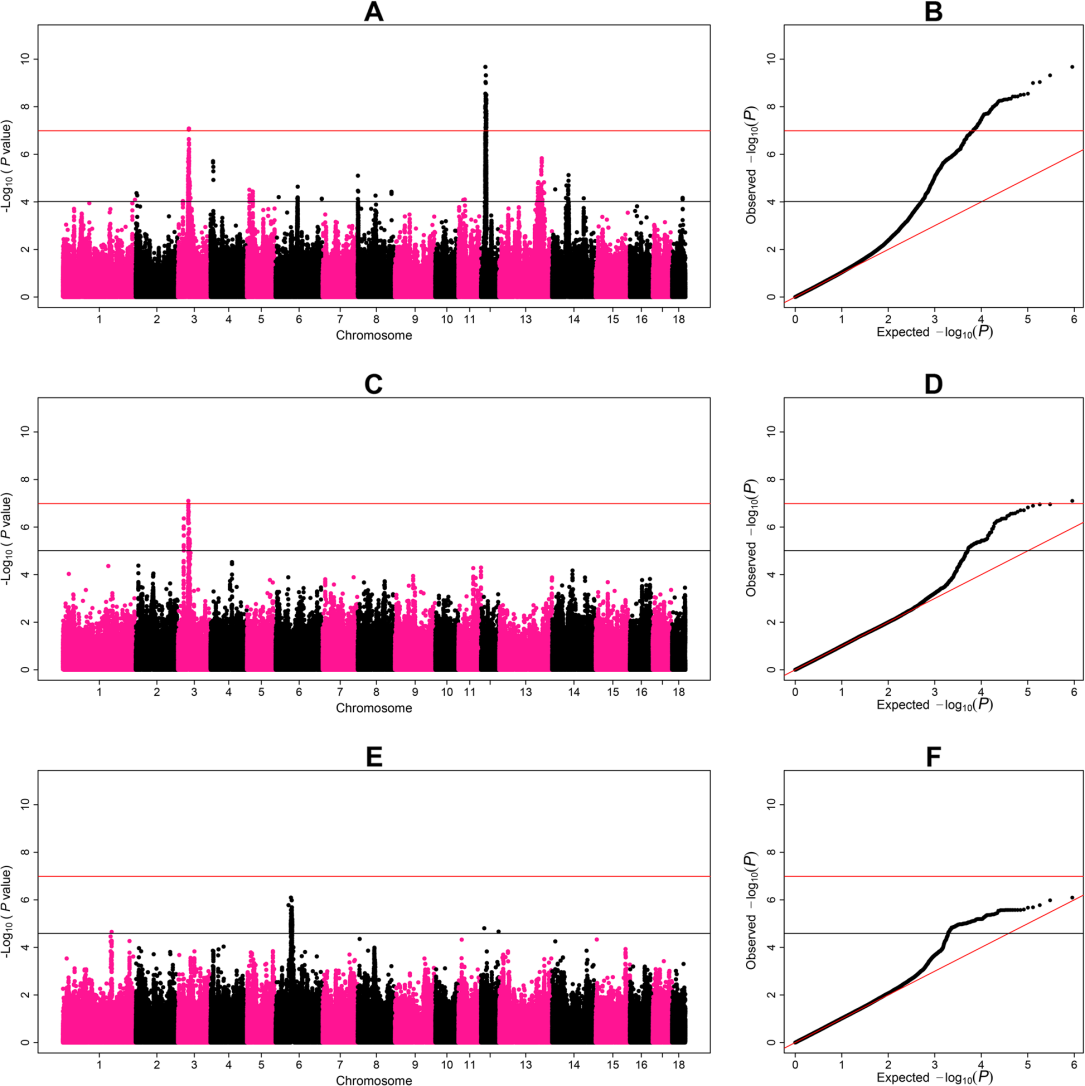
Genome-wide association study with the effect of SNPs on semen traits. On the *x*-axis is the chromosome position and on the *y*-axis is the −log_10_(*P*-value) of the association test for each SNP. The black line indicates the suggestive threshold based on a false discovery rate of 5%. The red line indicates the significance threshold based on the Bonferroni criterion. **A** and **B** The Manhattan plot and the QQ-plot for the additive effects of SNPs on abnormal sperm head. Two significant QTL can be seen on chromosomes SSC3 (38.5–44.1 Mb) and SSC12 (14.9–19.6 Mb). **C** and **D** The Manhattan plot and the QQ-plot for the dominance/recessive effects of SNPs on number of sperm in ejaculate. One significant QTL can be seen on chromosomes SSC3 (35.7–37.7 Mb). **E** and **F** The Manhattan plot and the QQ-plot for the dominance/recessive effects of SNPs on progressive motility of fresh semen. No significant QTL was detected in the GWAS

Manhattan and quantile-quantile plots (QQ-plot) of the GWAS for additive effects on abnormal head and dominance/recessive effects on number of sperm cells in ejaculate and progressive motility of fresh semen are in Fig. 1. Manhattan and QQ-plots for additive and dominance/recessive effects on all traits are in Additional file 1.

A table describing the position of QTL displaying significant additive and/or dominance/recessive effects on semen traits can be found in Additional file 2. For each QTL, effects of genotype classes and the percentage of phenotypic variance explained by their lead SNPs are in Table 2. In addition, a table summarizing QTL that did not reach the Bonferroni significance threshold but displayed suggestive effects on semen traits according to an FDR of 5% can be found in Additional file 3.

**Table 2:** Description of the effect of lead SNPs and SNP heritability

**Trait**	**Position**	***f***_***a***_^**a**^	**Genotypic effect**			${{\varvec{h}}}_{{\varvec{S}}{\varvec{N}}{\varvec{P}}}^{2}\boldsymbol{ }\left(\boldsymbol{\%}\right)$^**b**^	**Additive** **−log**_**10**_**(*P*-value)**^**c**^	**Dominance/recessive** **−log**_**10**_ **(*P*-value)**^**c**^
			${\varvec{A}}{\varvec{A}}$	${\varvec{A}}{\varvec{a}}$	${\varvec{a}}{\varvec{a}}$			
Concentration, ×10^6^/mL	SSC3: 43.2	0.47	4.5 _(1.7)_	0	−12.7 _(1.7)_	1.1	7.2	-
	SSC12: 7.6	0.03	−0.8 _(3.1)_	0	80.8 _(15.0)_	0.4	8.9	7.6
	SSC14: 105.4	0.07	3.3 _(2.2)_	0	68.4 _(13.9)_	0.4	-	7.8
Number of sperm cells, ×10^9^ cells	SSC3: 43.5	0.48	1.7 _(0.7)_	0	−5.3 _(0.7)_	1.2	7.3	-
	SSC3: 36.7	0.34	0.6 _(0.6)_	0	−5.9 _(0.7)_	1.1	-	7.1
Proximal cytoplasmic droplets, %	SSC14: 46.5	0.08	−0.9 _(0.2)_	0	6.3 _(0.6)_	1.9	8.6	-
Distal cytoplasmic droplets, %	SSC6: 63.7	0.33	0 _(0.1)_	0	1.1 _(0.2)_	1.0	-	7.3
Distal midpiece reflex, %	SSC2: 136.6	0.42	−0.5 _(0.2)_	0	1.4 _(0.2)_	3.9	12.4	-
Abnormal head, %	SSC3: 42.0	0.46	−0.1 _(0.1)_	0	0.3 _(0.1)_	0.7	7.1	-
	SSC12: 16.1	0.45	0.2 _(0.1)_	0	−0.2 _(0.1)_	0.5	9.7	-

The effects of each genotype class (β) was estimated by fitting the allele count as a fixed class effect in a post-GWAS analysis. AA = homozygotic for the major allele, Aa = heterozygotic and aa = homozygotic for the minor allele. The effects of genotype classes of lead SNPs were estimated on untransformed traits

^a^*f*_*a*_ = Allele frequency of the minor allele

^b^The percentage of variance explained by lead SNPs was calculated for untransformed semen quantity and transformed sperm morphology traits

^c^The *P*-value threshold was –log_10_(*P*-value) > 7.0. Non-significant estimates are indicated with a dash

We identified 10 QTL with significant effects on six different semen traits (Table 2). We defined QTL with additive effects if their lead SNPs were significant in the additive GWAS and QTL with dominance/recessive effects if their lead SNPs were significant in the dominance/recessive GWAS. Six QTL displayed only additive effects, three QTL displayed only dominance/recessive effects and one QTL displayed both additive and dominance/recessive effects. No significant QTL were identified for sperm motility traits (Fig. 1E). The size of the QTL regions ranged from 2.0 to 17.2 Mb, and included between 1 and 64 significantly associated SNPs according to the Bonferroni threshold (−log_10_(*P*-value) > 7.0). A region of particular interest was found on chromosome SSC3, where lead SNPs of four QTL were identified for concentration, number of sperm cells and abnormal head morphology within 7 Mb of proximity (i.e., 36.7–43.5 Mb) (Fig. 1A and C).

We focused further analyses on the lead SNPs of significant QTL. Lead SNPs explained between 0.4% and 3.9% of total phenotypic variance in particular semen traits. Most minor alleles of lead SNPs have deleterious effects on semen traits, i.e., decreased semen quantity or increased sperm morphological abnormalities. For example, boars homozygous for the minor allele of the lead SNP on locus SSC14 (46.5 Mb) produced semen with an increase of over 7% in proximal cytoplasmic droplets as compared to boars homozygous for the major allele (Table 2).

Minor alleles considered beneficial for reproductive success were present for lead SNPs on chromosomes SSC12 and SSC14. Although these were present at low frequencies, they showed increased ejaculate concentration. For example, the lead SNP on locus SSC12 (7.6 Mb) showed a recessive effect on increasing ejaculate concentration; boars homozygous for the minor recessive allele produced ejaculates with more 80 million sperm cells/mL than heterozygous or homozygous boars for the major dominant allele.

For loci with dominance/recessive effects, major alleles exerted complete dominance over minor alleles. This effect was consistent for both beneficial (e.g., locus SSC12 (7.6 Mb) associated with ejaculate concentration) and deleterious alleles (e.g., locus SSC6 (63.7 Mb) associated with distal cytoplasmic droplets).

### Genotype frequencies and effect of lead SNPs with age of the boar

The genotype frequencies and the effects of genotype classes for all lead SNPs at different ages were assessed (Table 3). For loci associated with semen quantity traits, the genotype frequencies of lead SNPs remained stable while the effect size increased with age of the boar. For example, from 7 to 13 months of age, boars homozygous for the deleterious allele on locus SSC3 (43.5 Mb) produced less 5.2 million sperm cells per ejaculate as compared to homozygous boars for the major allele. This effect increased with age: this difference was 7.9 million sperm cells at 14 to 23 months of age and 9.1 million sperm cells at 24 to 60 months of age.

**Table 3:** Genotypic frequencies and genotypic effects for lead SNPs with age of the boar

**Trait**	**Position**	**Age of the boar**	***f***_***AA***_	***f***_***Aa***_	***f***_***aa***_	${\varvec{A}}{\varvec{A}}$	${\varvec{A}}{\varvec{a}}$	${\varvec{a}}{\varvec{a}}$
Concentration, ×10^6^/mL	SSC3: 43.2	7–13 months	0.27	0.50	0.22	3.7 _(1.9)_	0	−12.2 _(2.0)_
		14–23 months	0.28	0.50	0.22	5.2 _(2.3)_	0	−13.1 _(2.4)_
		24–60 months	0.28	0.51	0.20	8.6 _(2.5)_	0	−10.4 _(2.6)_
	SSC12: 7.6	7–13 months	0.94	0.06	<0.01	2.4 _(3.5)_	0	54.4 _(16.1)_
		14–23 months	0.94	0.06	<0.01	0.4 _(4.1)_	0	74.6 _(18.3)_
		24–60 months	0.93	0.07	<0.01	−2.4 _(4.5)_	0	82.3 _(19.4)_
	SSC14: 105.4	7–13 months	0.86	0.14	<0.01	2.7 _(2.4)_	0	46.8 _(14.5)_
		14–23 months	0.86	0.14	<0.01	2.3 _(2.9)_	0	56.5 _(16.9)_
		24–60 months	0.86	0.14	<0.01	0.6 _(3.2)_	0	72.6 _(17.8)_
Number of sperm cells, 10^9^ cells	SSC3: 43.5	7–13 months	0.32	0.49	0.18	0.3 _(0.6)_	0	−4.8 _(0.7)_
		14–23 months	0.33	0.49	0.18	1.2 _(0.7)_	0	−6.7 _(0.8)_
		24–60 months	0.33	0.50	0.17	3.0 _(0.9)_	0	−6.1 _(1.0)_
	SSC3: 36.7	7–13 months	0.27	0.50	0.22	1.3 _(0.6)_	0	−4.3 _(0.6)_
		14–23 months	0.28	0.50	0.22	1.8 _(0.7)_	0	−5.7 _(0.8)_
		24–60 months	0.28	0.51	0.21	2.3 _(1.0)_	0	−6.5 _(1.1)_
Proximal cytoplasmic droplets, %	SSC14: 46.5	7–13 months	0.85	0.14	<0.01	−1.3 _(0.2)_	0	7.2 _(1.2)_
		14–23 months	0.86	0.14	<0.01	−0.8 _(0.2)_	0	4.1 _(1.2)_
		24–60 months	0.87	0.13	0	−0.4 _(0.3)_	0	-
Distal cytoplasmic droplets, %	SSC6: 63.7	7–13 months	0.43	0.47	0.10	0.1 _(0.1)_	0	0.8 _(0.1)_
		14–23 months	0.43	0.48	0.09	0.1 _(0.1)_	0	1.1 _(0.2)_
		24–60 months	0.41	0.50	0.09	−0.1 _(0.2)_	0	0.9 _(0.3)_
Distal midpiece reflex, %	SSC2: 136.6	7–13 months	0.29	0.53	0.19	−0.4 _(0.2)_	0	1.3 _(0.2)_
		14–23 months	0.31	0.52	0.17	−0.4 _(0.2)_	0	1.3 _(0.2)_
		24–60 months	0.36	0.49	0.15	−0.1 _(0.2)_	0	1.0 _(0.2)_
Abnormal head, %	SSC3: 42.0	7–13 months	0.29	0.50	0.21	−0.1 _(0.1)_	0	0.4 _(0.1)_
		14–23 months	0.29	0.50	0.20	−0.2 _(0.1)_	0	0.3 _(0.1)_
		24–60 months	0.30	0.52	0.18	−0.2 _(0.1)_	0	0.2 _(0.1)_
	SSC12: 16.1	7–13 months	0.31	0.49	0.20	0.3 _(0.1)_	0	−0.1 _(0.1)_
		14–23 months	0.31	0.49	0.20	0.2 _(0.1)_	0	−0.2 _(0.1)_
		24–60 months	0.29	0.50	0.21	0.2 _(0.1)_	0	−0.2 _(0.1)_

The effects of each genotype class (β) was estimated by fitting the allele count as a fixed categorical effect in a multivariate analysis for semen traits measured from boars between 7 and 13 months, 14 to 23 months and 24 to 60 months of age. The effects of genotype classes of lead SNPs were estimated on untransformed traits. *f*_*AA*_ = frequency of homozygotes for the major allele, *f*_*Aa*_ = frequency of heterozygotes and *f*_*aa*_ = frequency of homozygotes for the minor allele

For loci associated with sperm morphology traits, the frequency of homozygous boars for deleterious minor alleles slightly decreased while the effect size remained stable with age of the boar. For example, the frequency of boars homozygous for the deleterious allele in locus SSC2 (136.6 Mb) associated with distal midpiece reflex (%) was 0.19 for boars at 7 to 13 moths of age and 0.15 for boars at 24 to 60 months of age.

### Genetic trends for QTL

The allele frequencies per year and the regression coefficient of the minor allele frequency on year of birth of the boar for all lead SNPs are listed in Additional file 4.

**Fig. 2:**
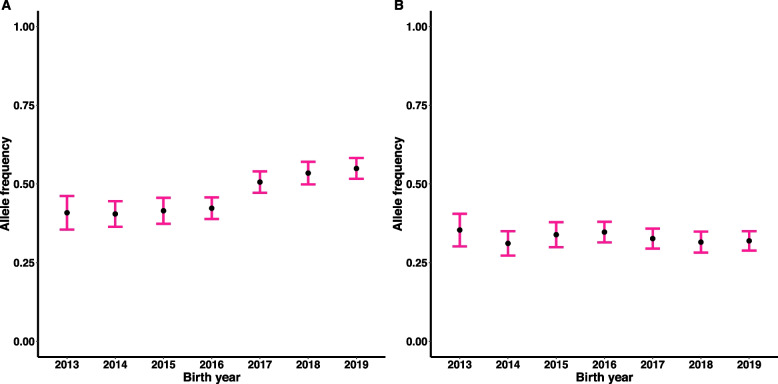
Changes in frequency of the minor allele for loci SSC3 (42.0 Mb) (**A**) and SSC6 (63.7 Mb) (**B**) associated with abnormal head morphology and distal cytoplasmic droplets, respectively

The frequency of some minor alleles with deleterious effects increased between 2013 and 2019. This pattern was observed for loci on chromosome SSC3 (36.5–43.5 Mb) and SSC2 (136.6 Mb). For example, in 2013 the minor allele of the locus SSC3 (42.0 Mb) (Fig. 2A) associated with increased abnormal head morphology was 0.39 and increased up to 0.56 in 2019, becoming the major allele. Some deleterious alleles which had a relatively low frequency, further decreased between 2013 and 2019. For example, in 2013 the minor allele of the locus SSC14 (46.5 Mb) associated with proximal cytoplasmic droplets was 0.14 and decreased to 0.10 by 2019. For two lead SNPs, SSC12 (16.1 Mb) and SSC6 (63.7 Mb) (Fig. 2B) with effects on proximal and distal cytoplasmic droplets, respectively, the minor allele frequency remained stable over time.

### Effects of lead SNPs on other semen traits

For lead SNPs showing an effect on multiple traits, the effects of genotype classes on all affected traits can be found in Additional file 5. Overall, most lead SNPs showed effects on several semen traits (−log_10_(*P*-value) > 3.0) with the exception of two loci on chromosome SSC12 (7.6 and 16.1 Mb), which were only significantly associated with either ejaculate concentration or abnormal head morphology, respectively.

For semen quantity traits, loci on chromosomes SSC3 (36.7, 42.0, 43.2 and 43.5 Mb) and SSC14: 105.4 Mb showed positive effects on both concentration and number of sperm cells. For the locus on chromosome SSC14 (105.4 Mb), boars homozygous for the minor allele produced ejaculates with an increase in both concentration and number of sperm cells.

For sperm morphology traits, loci on chromosomes SSC2 (136.6 Mb), SSC6 (63.7 Mb) and SSC14 (46.5 Mb) showed effects on several morphology traits. For example, boars homozygous for the minor allele at the locus on chromosome SSC14 produced ejaculates with an increase in proximal cytoplasmic droplets and also in total and distal cytoplasmic droplets.

For sperm motility traits, while no SNP reached the genome-wide significance threshold in the GWAS (−log_10_(*P*-value) > 7.0), two variants on chromosomes SSC2 (136.6 Mb) and SSC6 (63.7 Mb) associated with sperm morphological abnormalities also affected motility (−log_10_(*P*-value) > 3.0). For example, the locus on chromosome SSC6 was associated with distal cytoplasmic droplets in the GWAS and showed an effect on total and proximal cytoplasmic droplets, distal midpiece reflex, bent tail and total and progressive motilities of fresh and stored semen. Boars homozygous for the minor allele showed increased morphological abnormalities and decreased sperm motility.

### Functional analysis

The scores of the pCADD pipeline for WGS variants in high LD with lead SNPs of significant QTL are in Additional file 6, with candidate causal variants highlighted in purple. The subset of candidate causal variants, pCADD and SIFT scores and affected genes are in Table 4. Most of the GWAS signals could be linked to missense variants in a limited number of genes. Eight lead SNPs were in high LD with five candidate WGS missense variants. These WGS variants showed high pCADD scores (15.0–26.4) and low SIFT scores (0–0.11).

**Table 4:** Summary of pCADD results and candidate genes

**Trait**	**GWAS lead SNP**	**WGS SNP**	***r***^***2* a**^	**SNP annotation**	**pCADD score**	**SIFT score**	**Gene**
Concentration	SSC3: 43.2	SSC3: 40.1	0.87	Missense	20.2	-	*MEIOB*
	SSC12: 7.6	-	-	-	-	-	*-*
	SSC14: 105.4	-	-	-	-	-	-
Number of sperm cells	SSC3: 43.5	SSC3: 40.1	0.89	Missense	20.2	-	M*EIOB*
	SSC3: 36.7	SSC3: 40.1	0.94	Missense	20.2	-	*MEIOB*
Proximal cytoplasmic droplets	SSC14: 46.5	SSC14: 46.5	-	Missense	26.4	0	*GAS2L1*
Distal cytoplasmic droplets	SSC6: 63.7	SSC6: 64.0	0.95	Missense	15.0	0.01	*CFAP74*
Distal midpiece reflex	SSC2: 136.6	SSC2: 136.7	0.99	Missense	23.3	0.11	*UBE2B*
Abnormal head	SSC3: 42.0	SSC3: 40.1	0.96	Missense	20.2	-	*MEIOB*
	SSC12: 16.1	SSC12: 16.6	0.90	Missense	25.1	0.03	*EFCAB13*

^a^*r*^*2*^ measure of linkage disequilibrium

The lead SNP on chromosome SSC2 (136.6 Mb) associated with distal midpiece reflex was in high LD (*r*^*2*^ = 0.95) with a missense variant in the ubiquitin conjugating enzyme E2 B (*UBE2B*) gene. All lead SNPs on chromosome SSC3 (36.7–43.5 Mb) associated with concentration, number of sperm cells or abnormal head morphology were in high LD (*r*^*2*^ = 0.87–0.96) with the same WGS missense variant, in the *MEIOB* gene. The lead SNP on chromosome SSC6 (63.7 Mb) associated with distal cytoplasmic droplets was in high LD (*r*^*2*^ = 0.95) with a WGS missense variant in the *CFAP74* gene. The lead SNP on chromosome SSC12 (16.1 Mb) associated with abnormal head was in high LD (*r*^*2*^ = 0.90) with a WGS missense variant in the *EFCAB13* gene. Finally, the lead SNP on chromosome SSC14 (46.5 Mb) associated with proximal cytoplasmic droplets was present in both the 660 K and WGS data. The lead SNP was in the *GAS2L1* gene.

## Discussion

Decreasing male fertility presents challenges in both wild and domestic populations, particularly in populations with reduced genetic diversity. While many wild species have small population sizes or lack genetic data for large-scale genetic studies, the domestic pig offers a unique opportunity to study male reproductive at a large scale. In commercial populations, boars are consistently genotyped and subject to routine semen evaluation. The combination of genotyping and phenotyping of large pig populations can be used to pinpoint the genetic basis of male fertility in pigs and offer insights into conserved genetic mechanisms not accessible for less-characterized mammalian species.

In this study, we performed a large-scale GWAS for semen traits in a purebred pig line to: 1) identify loci with additive and dominance/recessive effects on semen traits; 2) estimate genotypic effects and the phenotypic variance explained by these loci; 3) estimate changes in genotype effects with age of the boar; 4) estimate changes in allele frequency over time; 5) identify effects of lead SNPs on other semen traits; and 6) identify candidate causal variants and genes using whole-genome sequence (WGS) data. We reported heritabilities between 0.04 and 0.28 and repeatabilities between 0.15 and 0.64 for semen traits. With GWAS, we identified a total of 10 QTL associated with six semen traits, of which four QTL showed dominance/recessive effect. We focused our analyses on the lead SNPs of these QTL. Lead SNPs explained between 0.4% and 3.9% of the phenotypic variance in semen traits with most minor alleles showing negative effects on these traits. The allele frequencies and the effect of these SNPs changed as boars matured (between 7 and 60 months of age) and the frequency of some deleterious alleles increased over time (2013–2019). We showed that most lead SNPs had effects on multiple semen traits and identified potential causal variants and genes based on pCADD scores for WGS variants.

### Genetic parameters

We estimated heritabilities and repeatabilities for semen traits using a repeatability model that accounted for relationships based on a genomic relationship matrix. Our results indicate that heritabilities ranged from 0.04 to 0.28, while repeatabilities ranged from 0.15 to 0.64 (for estimates, see Table 1). These estimates are in line with those from other studies [30, 31]. In the same population, we previously reported slightly higher heritabilities while using a pedigree-based relationship matrix [16, 25]. Differences in parameter estimates may be due to the use of a genomic relationship matrix as opposed to a pedigree-based relationship matrix [32]. In addition, not all animals in the population were genotyped and the number of animals included in the present study was smaller.

### QTL identification and functional annotation

We identified 10 QTL showing additive and/or dominance/recessive effects on male fertility. For each QTL, we estimated the effect size, examined allele frequency trends with age of the boar and over time, and integrated whole-genome sequencing data to identify likely causal variants. These results are presented and discussed by chromosome.

### QTL and functional analysis on chromosome SSC2

The lead SNP on chromosome SSC2 (136.6 Mb) displayed an additive effect on distal midpiece reflex (Table 2). The minor allele was deleterious: the increase in number of copies of the deleterious allele increased the percentage of distal midpiece reflex and to a lesser extent, distal cytoplasmic droplets and total morphological abnormalities and decreased total and progressive motility of fresh semen (Additional file 5). We observed that the frequency of heterozygous and homozygous boars for the deleterious allele slightly decreased with age of the boar (between 7 and 60 months of age) while the effect on distal midpiece reflex remained stable (Table 3). In this population, boars with low semen quality are taken out of semen production and culled, and only a subset of selected boars are present at older ages [16]. The decrease in frequency of carriers for this deleterious allele is consistent with the culling practices of AI centres. The lead SNP is in high LD with a WGS missense variant that has a high pCADD score and a very low SIFT score (Table 4). This variant is in the *UBE2B* gene. These observations suggest that the minor allele of the WGS variant likely disrupts the function of the *UBE2B* gene. This gene plays a role in post-replication DNA repair [33, 34] and assembly of the central scaffold of microtubules in the tail of sperm cells [35]. A previous study in mice showed that *UBE2B* knock-outs were infertile due to abnormal spermatogenesis [36]. In humans, studies have reported a set of variants that disrupted the expression of this gene, resulting in male infertility [37]. The increase in sperm morphological abnormalities and decrease in sperm motility are likely due to improper assembly of microtubules in the sperm tail. The frequency of this allele continuously increased between 2013 and 2019, a pattern that has been observed for alleles with positive effects and under selection but is not expected for alleles with deleterious effects [38]. Because drift produces random allele frequency changes, it is unlikely to explain the systematic increase observed. In this population, selection is applied for several traits, with a strong emphasis on production traits. This suggests that this locus could have an antagonistic pleiotropic effect or is located close to a locus affecting a production trait under selection. There have been several instances in which deleterious alleles increased rapidly in a population due to an antagonistic positive effect on a trait of interest [39, 40]. In this regard, the *UBE2B* gene has been described to promote protein degradation and muscle atrophy in skeletal muscle in pigs [41, 42]. Defective alleles in this gene may be beneficial for improving meat production traits. Future research should investigate the relationship between production and male reproductive traits in pigs.

### QTL and functional analysis on chromosome SSC3

**Fig. 3:**
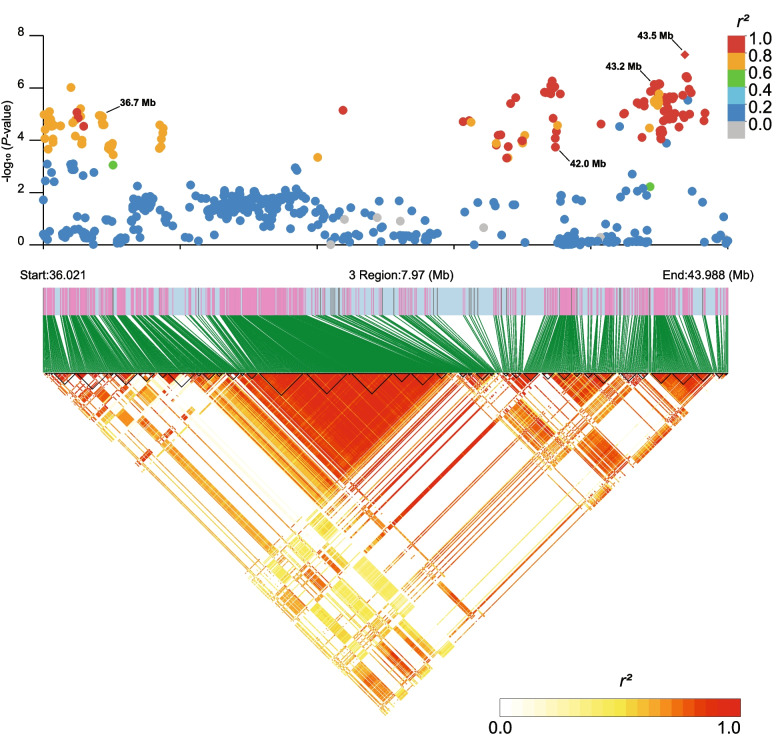
Additive effect of SNPs for number of sperm cells on chromosome SSC3 (36.0–44.0 Mb) and linkage disequilibrium with the lead SNP at SSC3: 43.5 Mb. The top panel shows the −log_10_(*P*-value) of SNPs from the GWAS for additive effects on number of sperm cells. Lead SNPs associated with abnormal sperm head morphology, concentration, and number of sperm cells are highlighted. SNPs are coloured according to their LD with the lead SNP. The bottom panel displays the LD block structure in the region. The plot was generated using LDBlockShow [43]

Lead SNPs on chromosome SSC3 (36.7–43.5 Mb) displayed effects on total number of sperm cells, ejaculate concentration and abnormal sperm head morphology. An increase in number of deleterious copies decreased the total number of sperm cells and ejaculate concentration and increased the percentage of sperm cells with abnormal head morphology. We observed that genotype frequencies did not change with age of the boar, likely because boars in this population are not culled due to low semen quantity [16]. All lead SNPs in this region were within 7 Mb of proximity and in high LD with the same WGS missense variant (SSC3: 40.1 Mb) with a high pCADD score. Additionally, we calculated pairwise LD for SNPs within this region. Figure 3 shows the −log_10_(*P*-values) of the GWAS for number of sperm cells, with SNPs coloured based on LD with the lead SNP and the LD blocks within the 7 Mb region.

While lead SNPs are not all located in the same LD block, pairwise LD between lead SNPs was high. This is evidence that the same missense variant is likely affecting these traits. The missense variant is located in the meiosis specific with OB-fold (*MEIOB*) gene and likely disrupts its function. The *MEIOB* gene is exclusively expressed in testis [44] and during meiosis, its proteins form a complex with the spermatogenesis associated 22 (*SPATA22*) proteins to coordinate normal meiotic recombination [45]. In mice, *MEIOB* knock-outs produced low sperm count [46]. In humans, several loss-of-function variants in the *MEIOB* gene have been described in azoospermic men, triggering gamete arrest and increasing apoptosis [47–49]. The decrease in total number of sperm cells and ejaculate concentration are likely caused by a disruption in meiotic recombination. The allele frequency of this deleterious allele increased between 2013 and 2019. Similar to the locus on chromosome SSC2, a consistent increase in allele frequency is unlikely to result from genetic drift, which produces random changes in allele frequency. Because the *MEIOB* gene is unlikely to influence production traits due to its exclusive expression in testis, this locus is likely located close to a locus affecting a production trait under selection. In this regard, a missense variant in the interleukin-1-alpha (*IL1A*) gene was in high LD with lead SNPs in this region and the *IL1A* gene has been extensively studied for its role in intramuscular fat content and fatty acid composition in pigs [50, 51]. It is possible that selection for *IL1A* alleles beneficial for meat production is increasing the frequency of the deleterious allele for male reproduction in the *MEIOB* gene due to a hitchhiking effect. Future work is necessary to study this genomic region and how it is affected by selection.

### QTL and functional analysis on chromosomes SSC6, SSC12 and SSC14

A lead SNP on chromosome SSC6 (63.7 Mb) displayed a dominance/recessive effect on distal cytoplasmic droplets and, to a smaller extent, on all other sperm tail morphology and motility traits (Additional file 5). The minor allele was recessive and deleterious: an increase in sperm tail abnormalities and a decrease in sperm motility was only evident in boars carrying two copies of the deleterious allele. The frequency of recessive homozygous boars slightly decreased with age, likely due to culling practices for boars with low semen quality [16]. The lead SNP was in high LD with a WGS missense variant in the cilia and flagella associated protein 74 (*CFAP74*) gene. This gene plays a role in the structural assembly of sperm flagella [52], and deleterious variants in *CFAP74* have been implicated in multiple morphological abnormalities of the sperm flagella in humans [53]. The increase in sperm tail abnormalities and decrease in sperm motility are likely caused by improper assembly of the sperm flagella, a condition which is only evident when both alleles of the *CFAP74* gene are defective. Regardless, the frequency of the deleterious allele remained stable between 2013 and 2019. In heterozygous individuals, recessive deleterious alleles are masked from (natural) selection by a dominant non-deleterious allele, increasing the probability for these alleles to persist in the population [54].

On chromosome SSC12, a lead SNP displayed an effect on ejaculate concentration (7.6 Mb) and this association was captured by both models (additive and dominance/recessive). The minor allele was recessive and beneficial: homozygous boars for this allele displayed increased ejaculate concentration. This allele was present at a low frequency and in a few homozygous boars (*n* = 8). We traced the origin of the beneficial alleles in the pedigree to understand if all homozygous boars originated from the same family. These alleles were scattered in the pedigree and the birth years of homozygous boars varied between 2006 and 2020. Based on the resolution in the pedigree, we could trace homozygotes to two major families and their founder homozygotes seemed to be unrelated. An examination on the frequency of collection of semen samples indicated that these boars were collected more often than an average AI boar, with a couple of homozygous boars being collected twice as many times per month as an average boar. It is likely that these boars were preferred in AI stations due to the increased number of semen doses that could be produced from a single ejaculate. The allele frequency of the minor allele remained low in the population and did not change between 2013 and 2019. In this case, it is possible that the beneficial allele remained undetected in the population, making it difficult to increase in frequency. Nevertheless, increasing the frequency of homozygous boars for this beneficial allele may result in an increased number of commercial semen doses being produced per ejaculate. Moreover, we did not identify a compelling candidate WGS variant. The majority of variants in high LD with the lead SNP were located in a gene that lacked annotation in the pig genome (ENSSSCG00000046071). Its human orthologue is the sidekick cell adhesion 2 (*SDK2*) gene which is highly expressed in testis [55]. Future research is necessary to determine the specific role of *SDK2* gene during spermatogenesis.

A lead SNP on chromosome SSC14 (46.5 Mb) displayed an additive effect on proximal cytoplasmic droplets and smaller effects on total and distal cytoplasmic droplets (Additional file 5). The minor allele was deleterious and increased the occurrence of cytoplasmic droplets. The heterozygous and homozygous for this allele decreased in frequency with age, with no homozygous boars present at older ages, likely due to culling [16]. The lead SNP was in high LD with a WGS missense variant in the growth arrest-specific 2 like 1 (*GAS2L1*) gene with a role in the assembly of the axoneme structure of the tail in sperm cells [56]. In mice, knock-out individuals produced sperm cells with hairpin-bend tail abnormalities [56]. The disruption of the *GAS2L1* gene likely alters the axoneme structure in sperm tails, preventing the removal of cytoplasmic droplets during spermatogenesis.

## Conclusions

We identified several loci with recessive and/or deleterious alleles for semen traits, accounting for a substantial proportion of phenotypic variation in these traits. The allele frequencies of some loci changed as boars aged likely due to culling. Some of these loci affected multiple semen traits and seemed to be in high LD with potential causal variants located in key genes involved in spermatogenesis (i.e., *MEIOB*) and sperm maturation (i.e., *CFAP74* and *UBE2B*). The frequency of a few deleterious alleles increased in the population between 2013 and 2019. The information from this study provides insight into genes affecting semen traits and can be used to select against deleterious alleles affecting these traits in pigs.

Our results contribute to narrowing the gap between genotype and phenotype by linking specific genetic variants to well-defined male fertility traits. These findings not only advance the understanding of male fertility in pigs but also have broader relevance to mammalian and human reproduction, where homologous genes are known to play similar roles in spermatogenesis. Moreover, this knowledge can inform conservation strategies in small, endangered populations where male fertility is a critical concern.

## Supplementary Information

Additional file 1. Manhattan plot and QQ-plot for the genome-wide association study of additive and dominance/recessive effects on semen traits. In the Manhattan plot, on the *x*-axis is the chromosome position and on the *y*-axis is the −log_10 _of the association test for each SNP. The black line indicates the suggestive threshold based on a false discovery rate of 5%. The red line indicates the significance threshold based on the Bonferroni criterion. The QQ-plot shows the expected versus observed distribution of the −log_10_.

Additional file 2. Summary of QTL significantly associated with semen traits. This table describes the positions of significant QTL, number of significant SNPs and the −log_10(_ (*P*-value) of lead SNPs.

Additional file 3. Summary of suggestive QTL associated with semen traits. Description: This table describes the positions of suggestive QTL according to a false discovery rate of 5%, the number of suggestive SNPs and the −log_10_(*P*-value) of lead SNPs.

Additional file 4. Estimates of the minor allele frequency of lead SNPs over time.

Additional file 5. The effect of lead SNPs on all semen traits.

Additional file 6. Results of the pCADD pipeline on WGS variants in LD with the significant lead SNPs.

## Data Availability

The dataset analysed in this study is property of Topigs Norsvin Research Center B.V. The dataset is available from Claudia A. Sevillano (claudia.sevillano@topigsnorsvin.com) upon reasonable request.

## Abbreviations

AI: Artificial insemination
CASA: Computer-assisted sperm analysis
GWAS: Genome-wide association study
LD: Linkage disequilibrium
QTL: Quantitative trait locus
SNP: Single nucleotide polymorphism
WGS: Whole-genome sequencing
