# Supplementary material for: A genome-wide association study reveals additive and recessive alleles affecting male fertility in pigs
Source: J Anim Sci Biotechnol. 2025 Dec 15;16:171. doi: 10.1186/s40104-025-01312-8 (PMC12703936; doi:10.1186/s40104-025-01312-8)
Supplement: Supplementary file 1 — Additional file 1. Manhattan plot and QQ-plot for the genome-wide association study of additive and dominance/recessive effects on semen traits. In the Manhattan plot, on the x-axis is the chromosome position and on the y-axis is the −log10 of the association test for each SNP. The black line indicates the suggestive threshold based on a false discovery rate of 5%. The red line indicates the significance threshold based on the Bonferroni criterion. The QQ-plot shows the expected versus observed distribution of the −log10. [file 40104_2025_1312_MOESM1_ESM.docx]

**
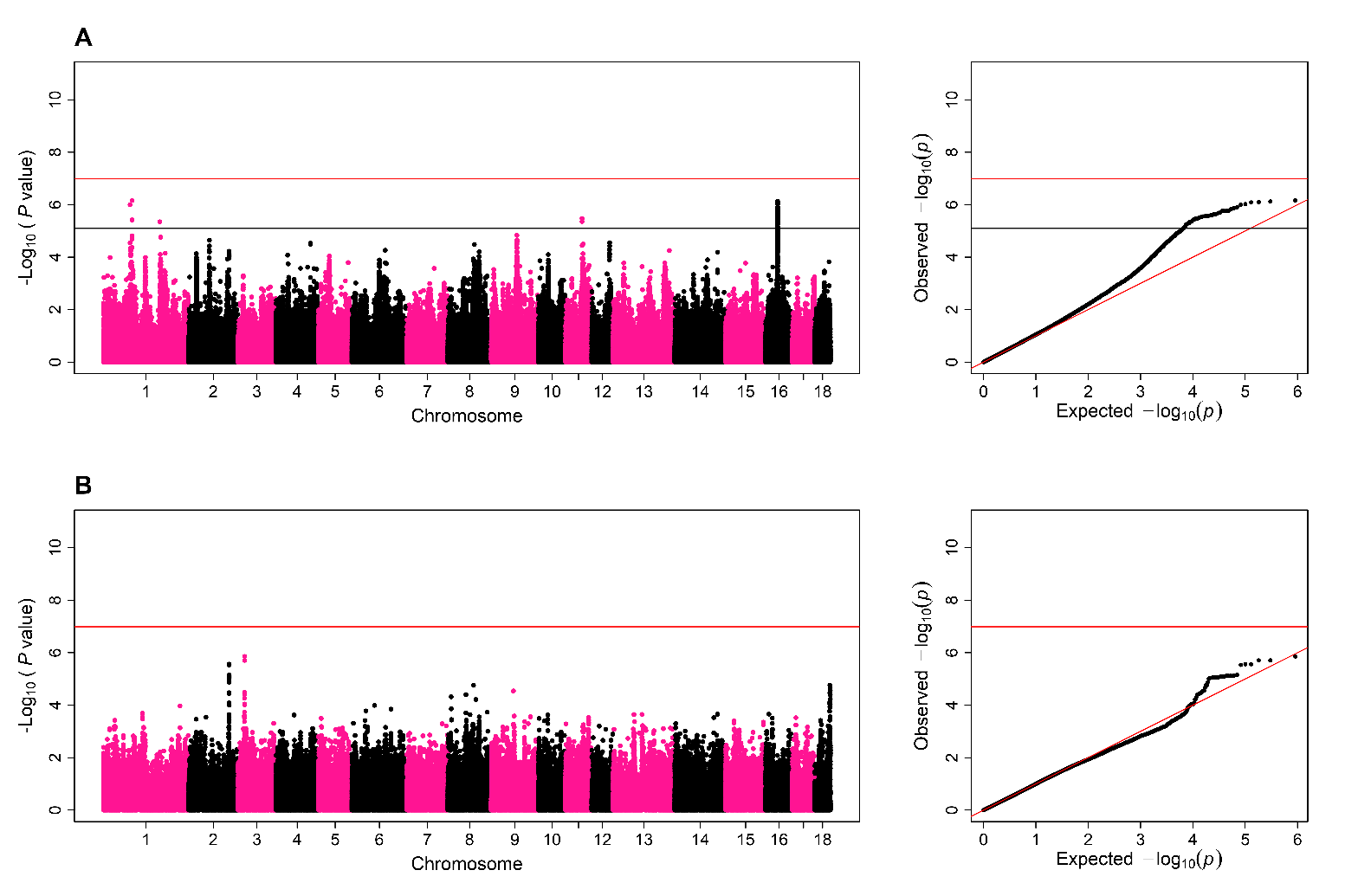
**

**Manhattan plot and QQ-plot for the genome-wide association study of additive (A) and dominance/recessive (B) effects on ejaculate volume.** In the Manhattan plots of the GWAS for both additive and dominance/recessive effects, each dot represents a SNP. The *x*-axis shows the chromosome position of SNP and the *y*-axis shows -log_10_(*P*-values) for the association test. The red line shows the threshold for significant associations according to the Bonferroni criterion. The black line shows the threshold for suggestive associations according to a false discovery rate of 5%. In the QQ-plot of the GWAS for both additive and dominance/recessive effects, each dot also represents a SNP but the *x*-axis and *y*-axis show the expected and observed distribution of −log_10_(*P*-values), respectively.

**
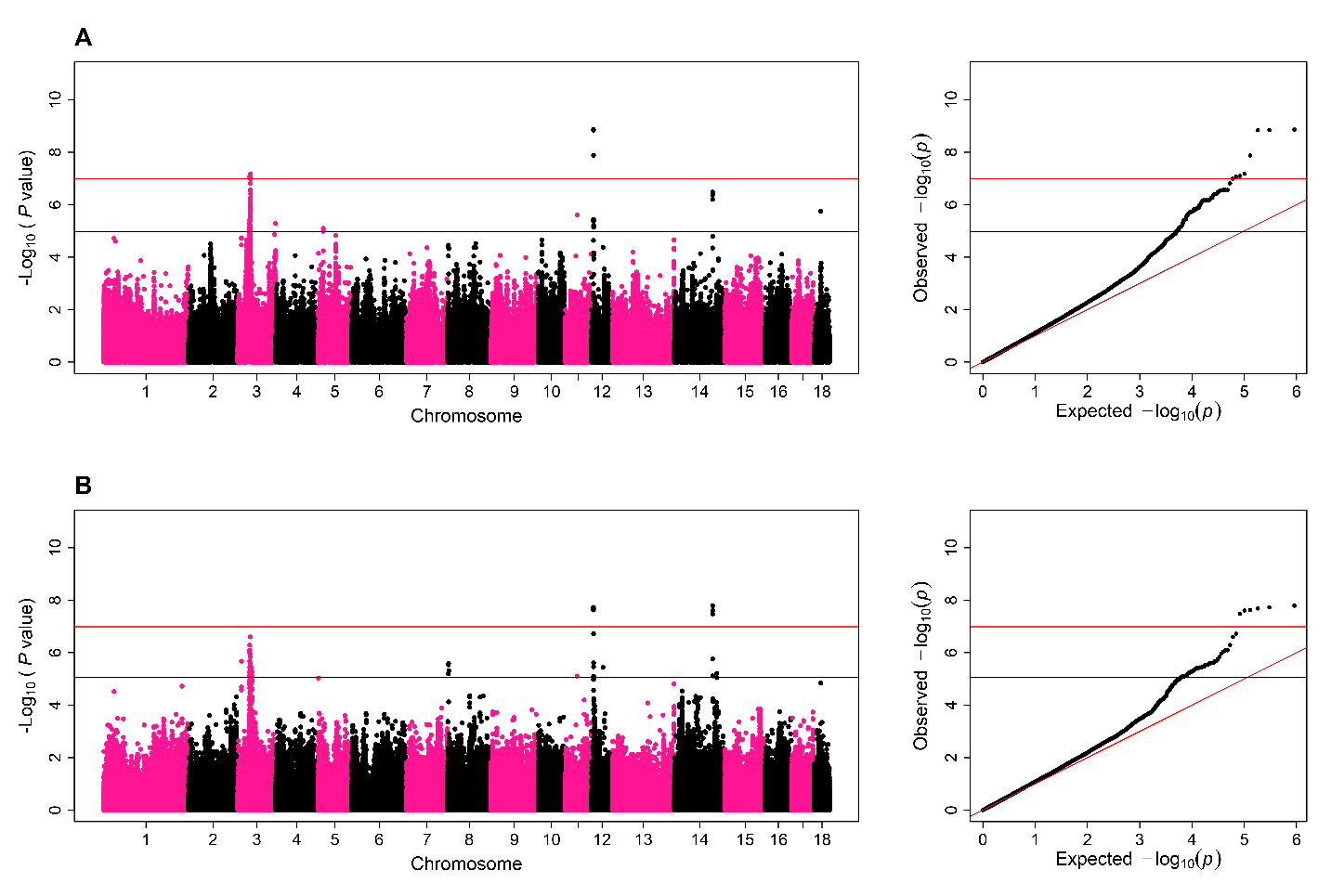
**

**Manhattan plot and QQ-plot for the genome-wide association study of additive (A) and dominance/recessive (B) effects on ejaculate concentration.** In the Manhattan plots of the GWAS for both additive and dominance/recessive effects, each dot represents a SNP. The *x*-axis shows the chromosome position of SNP and the *y*-axis shows −log_10_(*P*-values) for the association test. The red line shows the threshold for significant associations according to the Bonferroni criterion. The black line shows the threshold for suggestive associations according to a false discovery rate of 5%. In the QQ-plot of the GWAS for both additive and dominance/recessive effects, each dot also represents a SNP but the *x*-axis and *y*-axis show the expected and observed distribution of −log_10_(*P*-values), respectively.

**
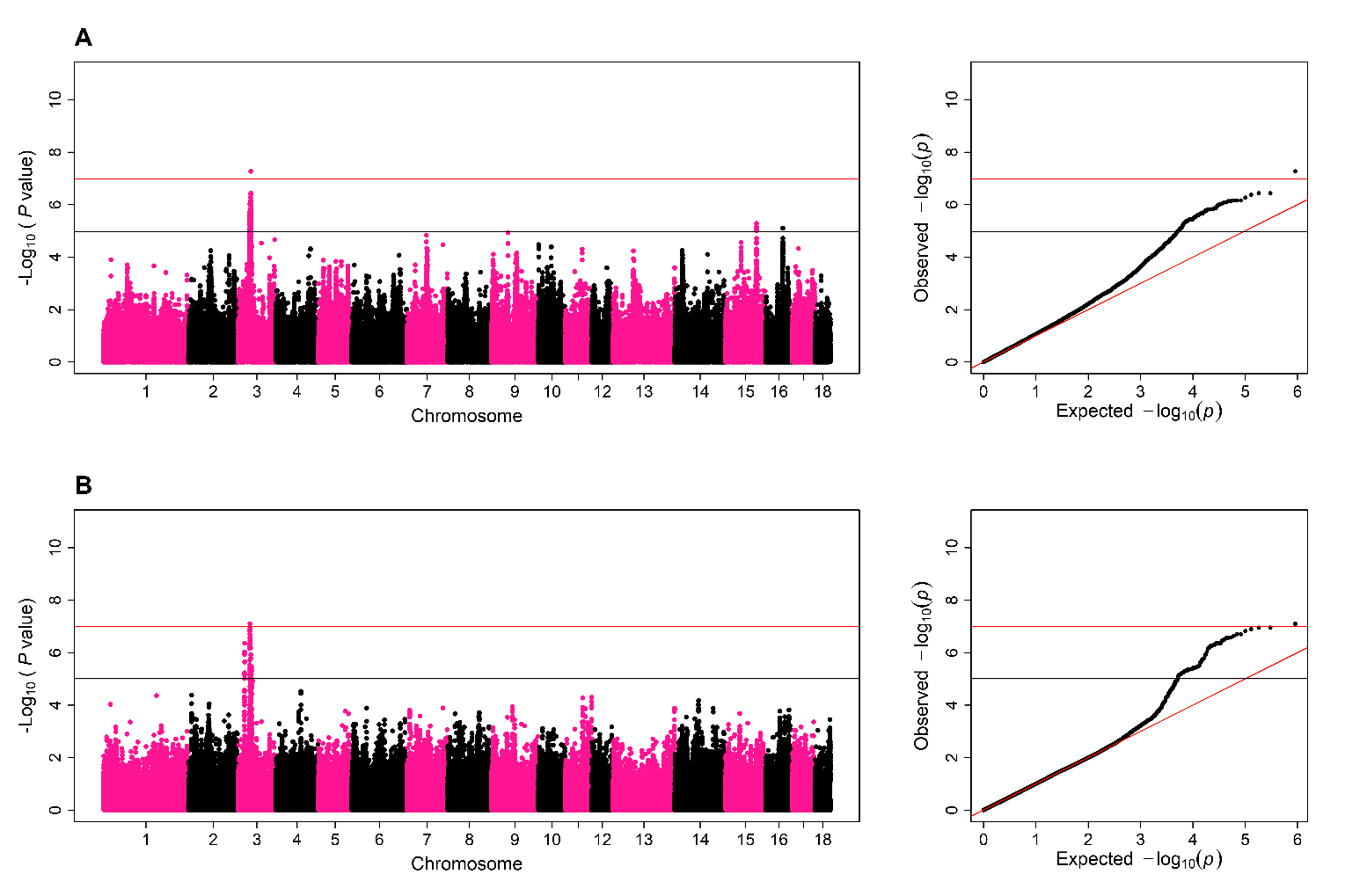
**

**Manhattan plot and QQ-plot for the genome-wide association study of additive (A) and dominance/recessive (B) effects on total number of sperm cells in an ejaculate.** In the Manhattan plots of the GWAS for both additive and dominance/recessive effects, each dot represents a SNP. The *x*-axis shows the chromosome position of SNP and the *y*-axis shows −log_10_(*P*-values) for the association test. The red line shows the threshold for significant associations according to the Bonferroni criterion. The black line shows the threshold for suggestive associations according to a false discovery rate of 5%. In the QQ-plot of the GWAS for both additive and dominance/recessive effects, each dot also represents a SNP but the *x*-axis and *y*-axis show the expected and observed distribution of −log_10_(*P*-values), respectively.

**
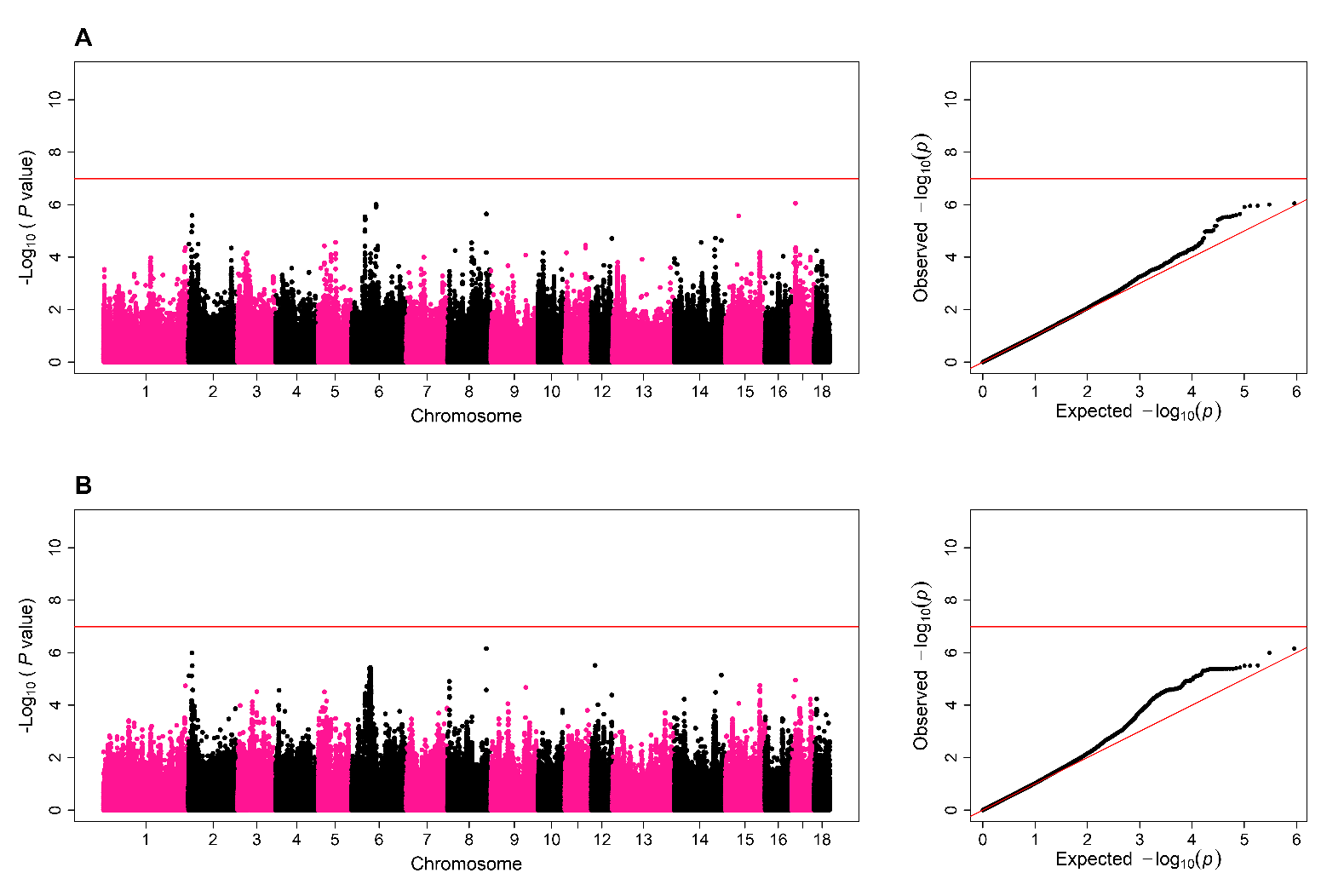
**

**Manhattan plot and QQ-plot for the genome-wide association study of additive (A) and dominance/recessive (B) effects on total motility of fresh semen.** In the Manhattan plots of the GWAS for both additive and dominance/recessive effects, each dot represents a SNP. The *x*-axis shows the chromosome position of SNP and the *y*-axis shows −log_10_(*P*-values) for the association test. The red line shows the threshold for significant associations according to the Bonferroni criterion. The black line shows the threshold for suggestive associations according to a false discovery rate of 5%. In the QQ-plot of the GWAS for both additive and dominance/recessive effects, each dot also represents a SNP but the *x*-axis and *y*-axis show the expected and observed distribution of −log_10_(*P*-values), respectively.

**
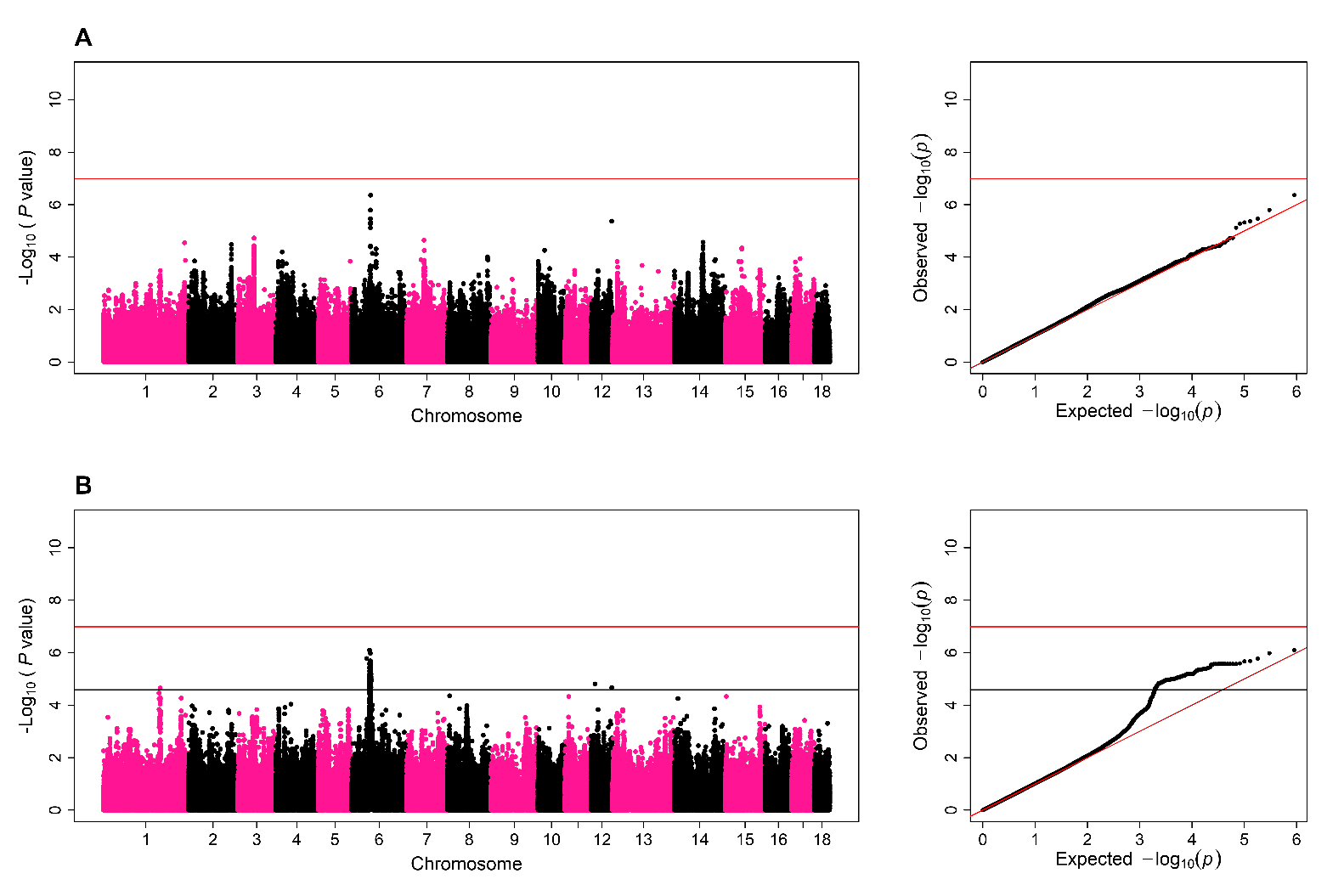
**

**Manhattan plot and QQ-plot for the genome-wide association study of additive (A) and dominance/recessive (B) effects on progressive motility of fresh semen.** In the Manhattan plots of the GWAS for both additive and dominance/recessive effects, each dot represents a SNP. The *x*-axis shows the chromosome position of SNP and the *y*-axis shows −log_10_(*P*-values) for the association test. The red line shows the threshold for significant associations according to the Bonferroni criterion. The black line shows the threshold for suggestive associations according to a false discovery rate of 5%. In the QQ-plot of the GWAS for both additive and dominance/recessive effects, each dot also represents a SNP but the *x*-axis and *y*-axis show the expected and observed distribution of −log_10_(*P*-values), respectively.

**
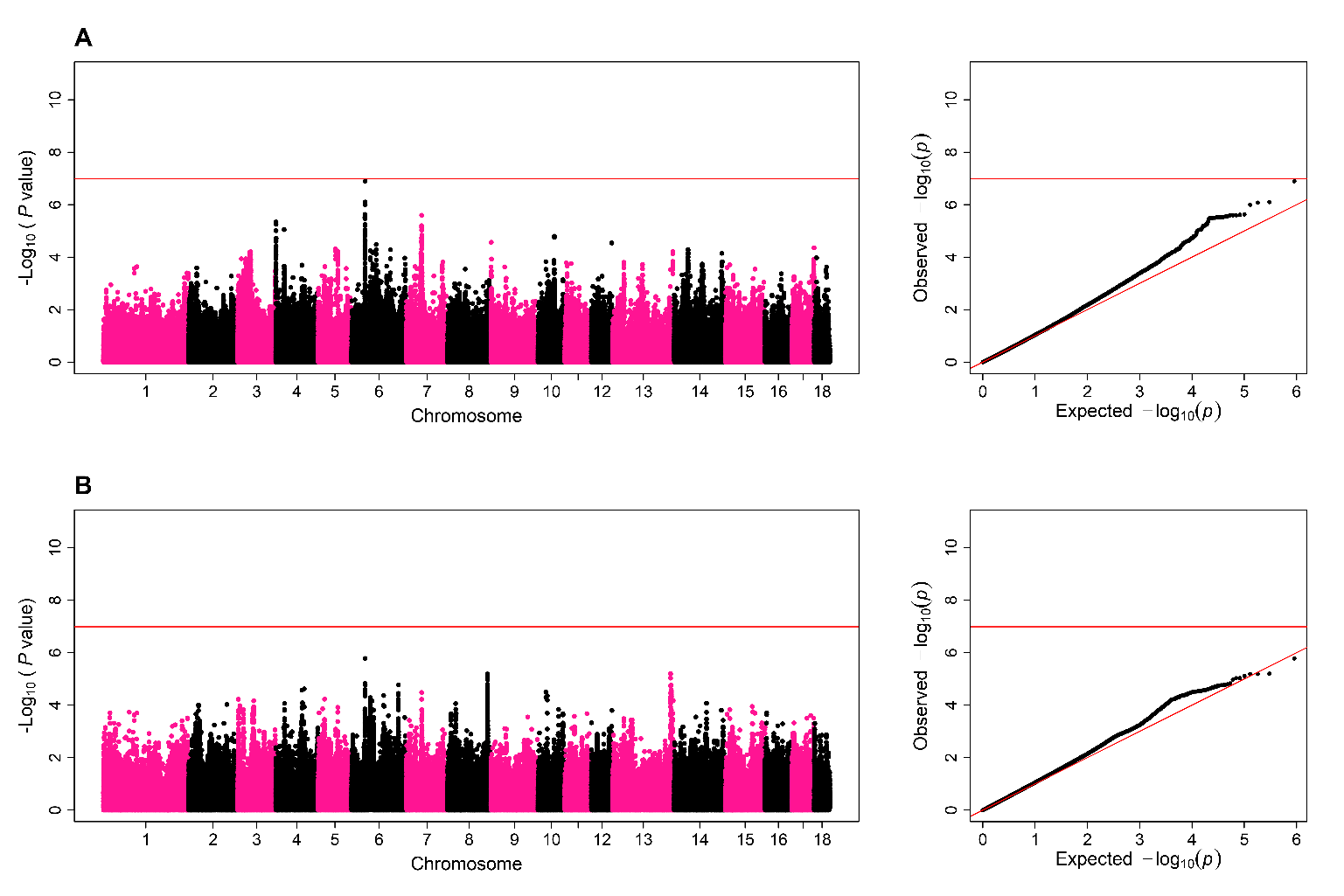
**

**Manhattan plot and QQ-plot for the genome-wide association study of additive (A) and dominance/recessive (B) effects on total motility after three days of storage.** In the Manhattan plots of the GWAS for both additive and dominance/recessive effects, each dot represents a SNP. The *x*-axis shows the chromosome position of SNP and the *y*-axis shows −log_10_(*P*-values) for the association test. The red line shows the threshold for significant associations according to the Bonferroni criterion. The black line shows the threshold for suggestive associations according to a false discovery rate of 5%. In the QQ-plot of the GWAS for both additive and dominance/recessive effects, each dot also represents a SNP but the *x*-axis and *y*-axis show the expected and observed distribution of −log_10_(*P*-values), respectively.

**
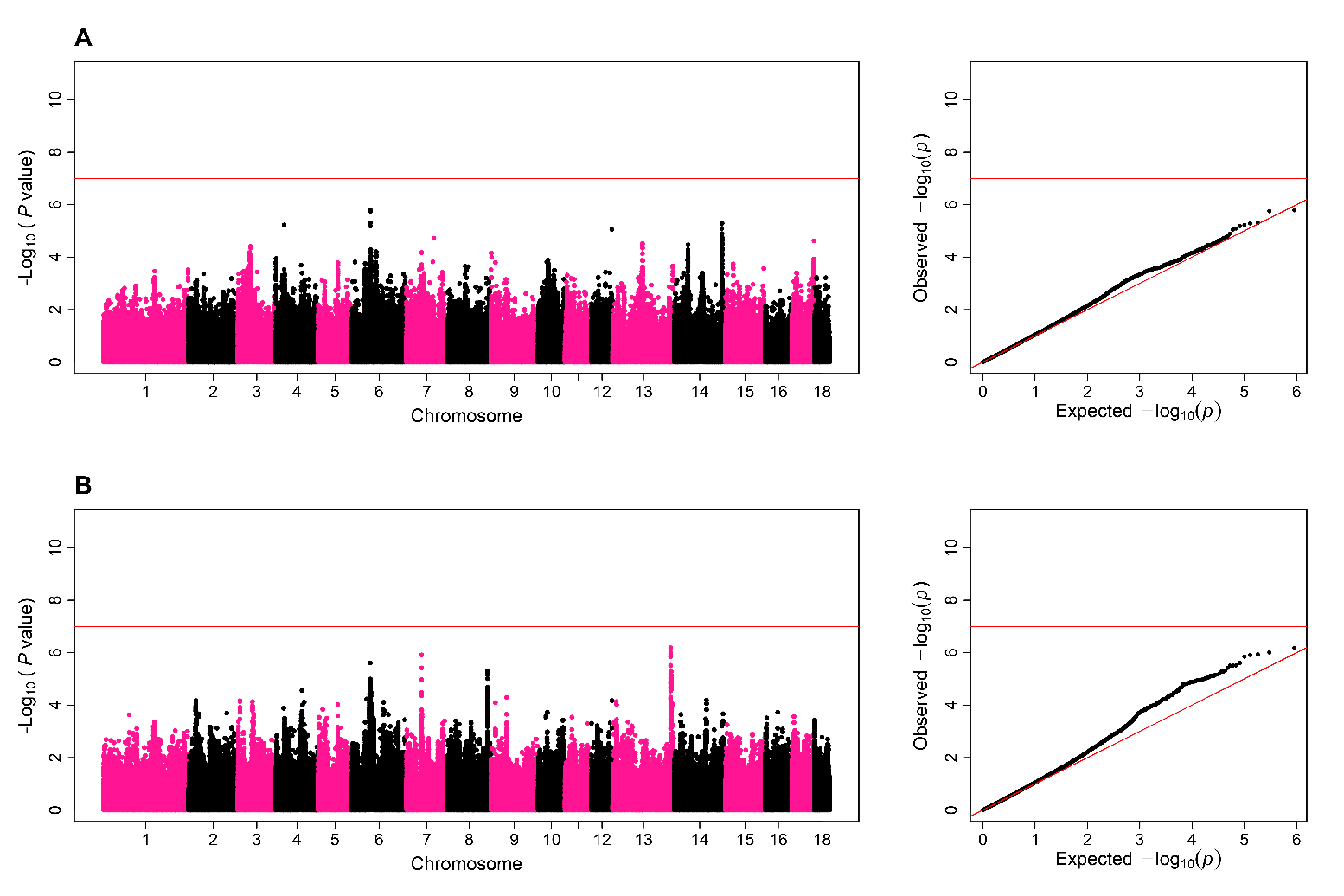
**

**Manhattan plot and QQ-plot for the genome-wide association study of additive (A) and dominance/recessive (B) effects on progressive motility after three days of storage.** In the Manhattan plots of the GWAS for both additive and dominance/recessive effects, each dot represents a SNP. The *x*-axis shows the chromosome position of SNP and the *y*-axis shows −log_10_(*P*-values) for the association test. The red line shows the threshold for significant associations according to the Bonferroni criterion. The black line shows the threshold for suggestive associations according to a false discovery rate of 5%. In the QQ-plot of the GWAS for both additive and dominance/recessive effects, each dot also represents a SNP but the *x*-axis and *y*-axis show the expected and observed distribution of −log_10_(*P*-values), respectively.

**
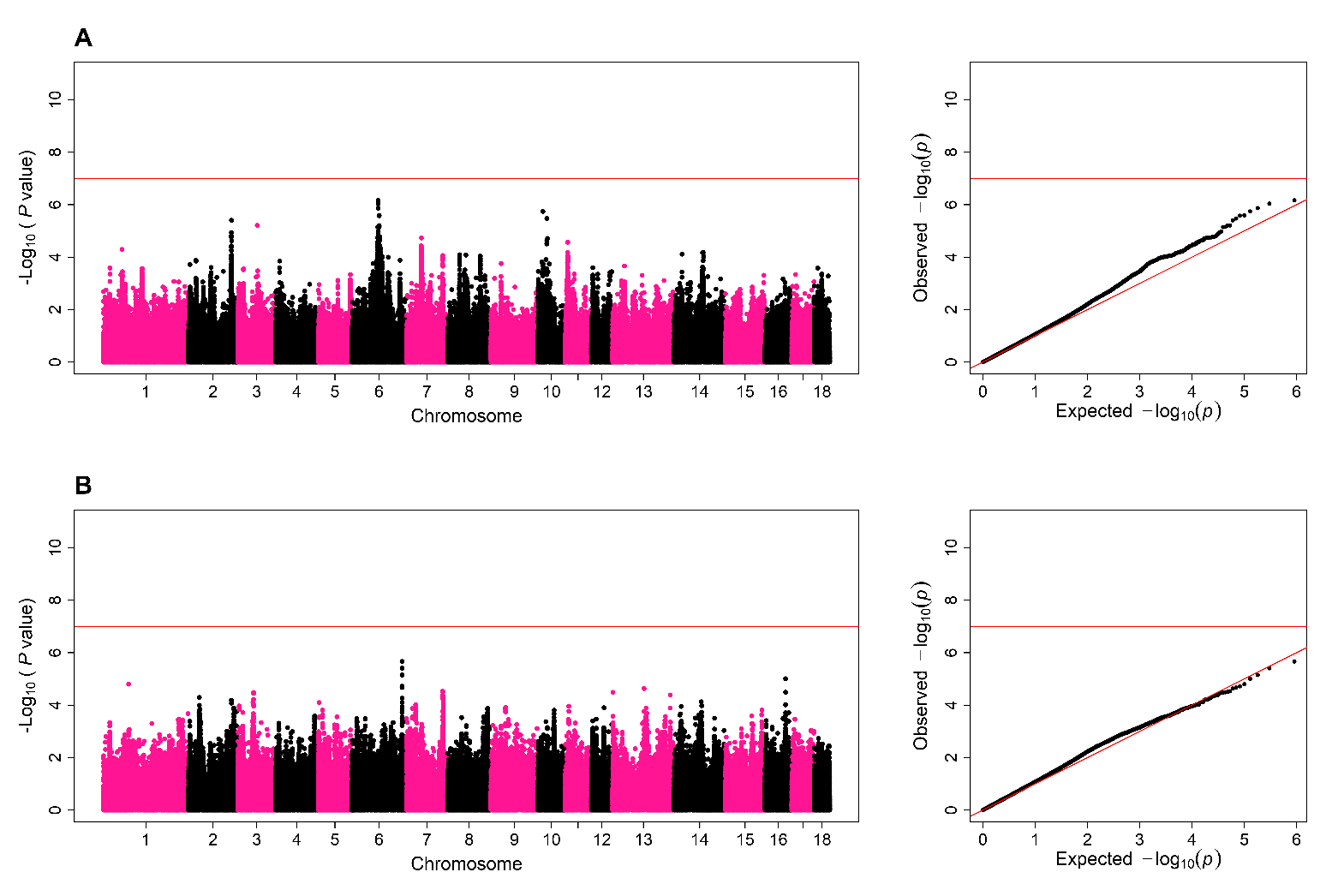
**

**Manhattan plot and QQ-plot for the genome-wide association study of additive (A) and dominance/recessive (B) effects on total morphological abnormalities.** In the Manhattan plots of the GWAS for both additive and dominance/recessive effects, each dot represents a SNP. The *x*-axis shows the chromosome position of SNP and the *y*-axis shows −log_10_(*P*-values) for the association test. The red line shows the threshold for significant associations according to the Bonferroni criterion. The black line shows the threshold for suggestive associations according to a false discovery rate of 5%. In the QQ-plot of the GWAS for both additive and dominance/recessive effects, each dot also represents a SNP but the *x*-axis and *y*-axis show the expected and observed distribution of −log_10_(*P*-values), respectively.

**
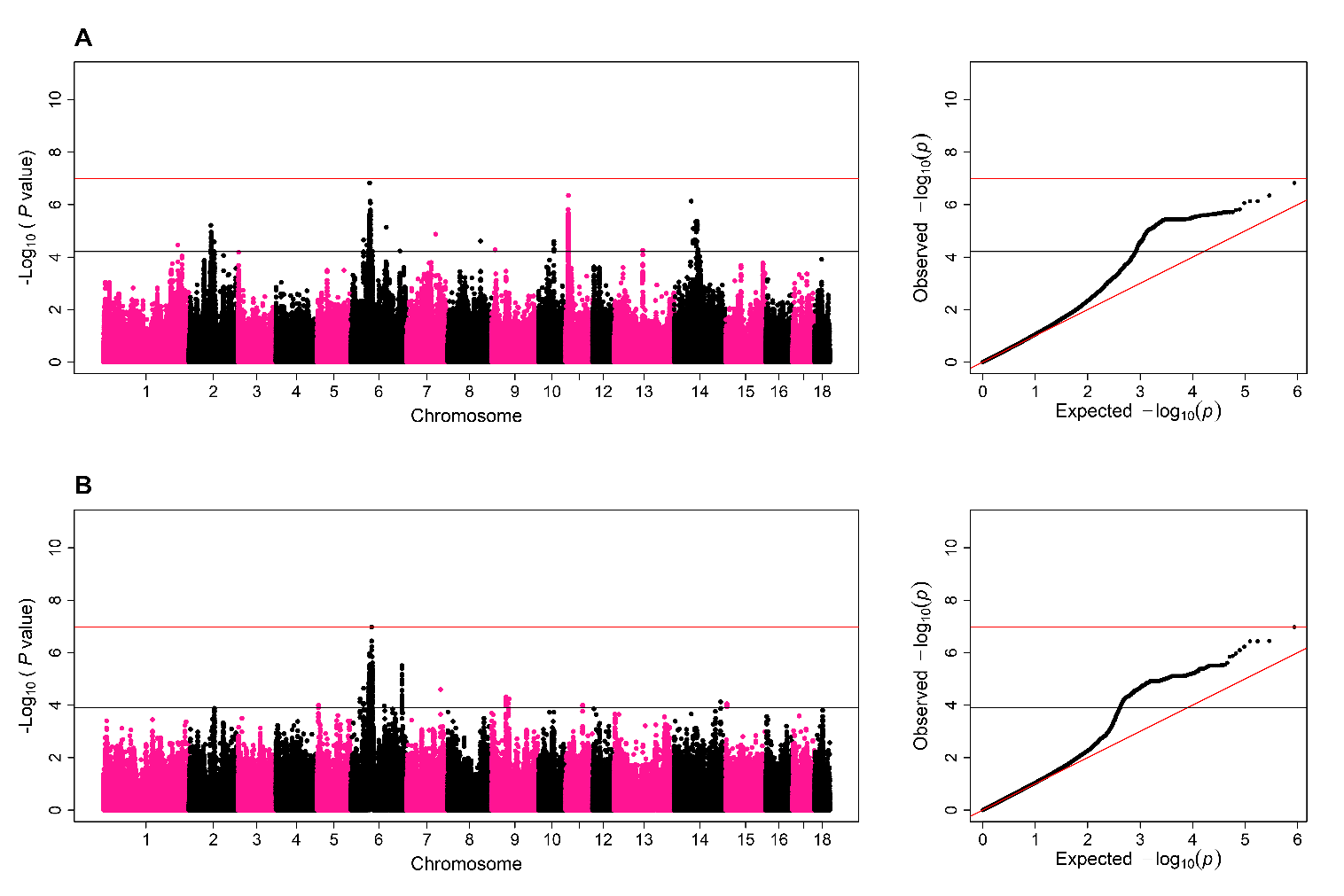
**

**Manhattan plot and QQ-plot for the genome-wide association study of additive (A) and dominance/recessive (B) effects on total cytoplasmic droplets.** In the Manhattan plots of the GWAS for both additive and dominance/recessive effects, each dot represents a SNP. The *x*-axis shows the chromosome position of SNP and the *y*-axis shows −log_10_(*P*-values) for the association test. The red line shows the threshold for significant associations according to the Bonferroni criterion. The black line shows the threshold for suggestive associations according to a false discovery rate of 5%. In the QQ-plot of the GWAS for both additive and dominance/recessive effects, each dot also represents a SNP but the *x*-axis and *y*-axis show the expected and observed distribution of −log_10_(*P*-values), respectively.

**
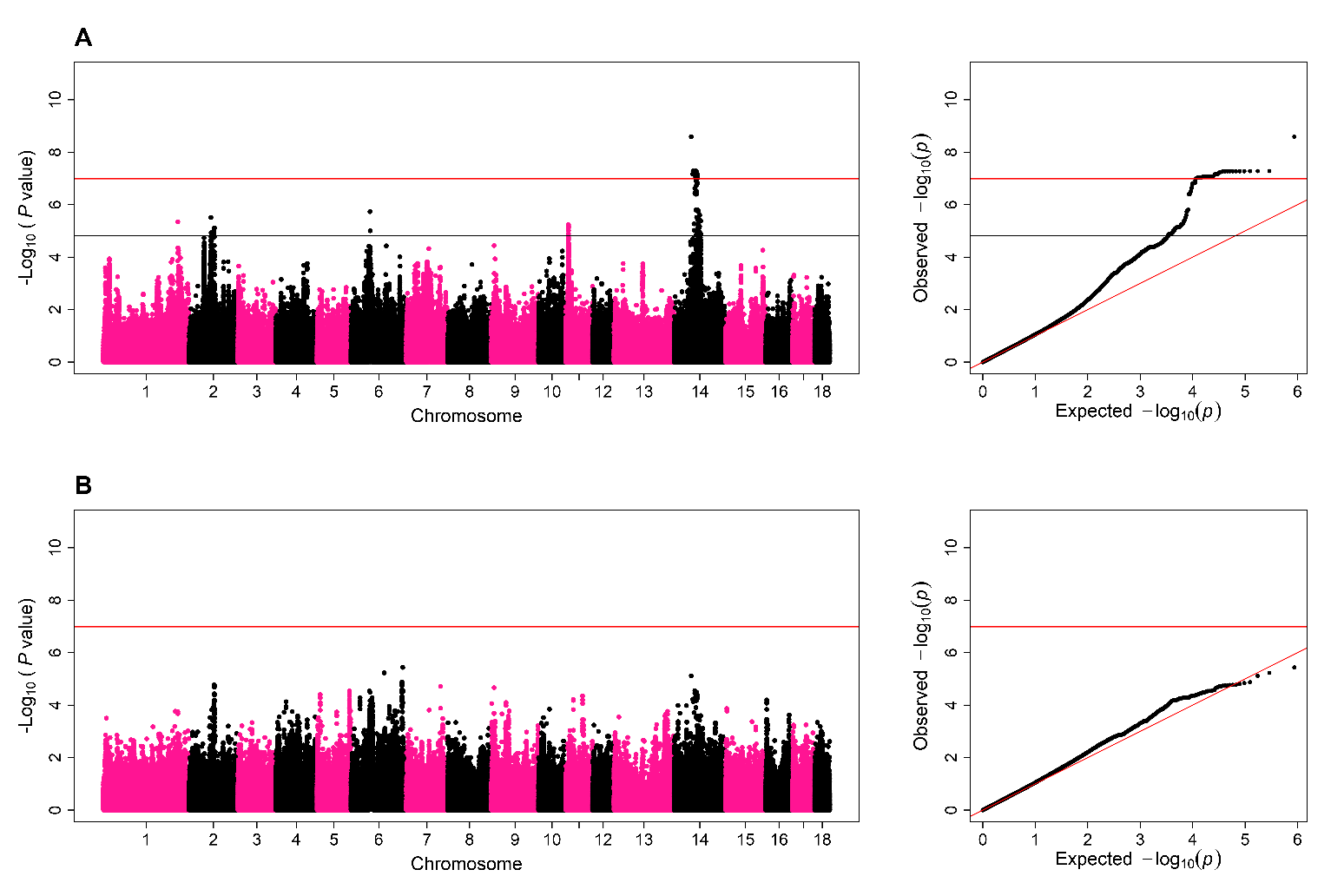
**

**Manhattan plot and QQ-plot for the genome-wide association study of additive (A) and dominance/recessive (B) effects on proximal cytoplasmic droplets.** In the Manhattan plots of the GWAS for both additive and dominance/recessive effects, each dot represents a SNP. The *x*-axis shows the chromosome position of SNP and the *y*-axis shows −log_10_(*P*-values) for the association test. The red line shows the threshold for significant associations according to the Bonferroni criterion. The black line shows the threshold for suggestive associations according to a false discovery rate of 5%. In the QQ-plot of the GWAS for both additive and dominance/recessive effects, each dot also represents a SNP but the *x*-axis and *y*-axis show the expected and observed distribution of −log_10_(*P*-values), respectively.

**
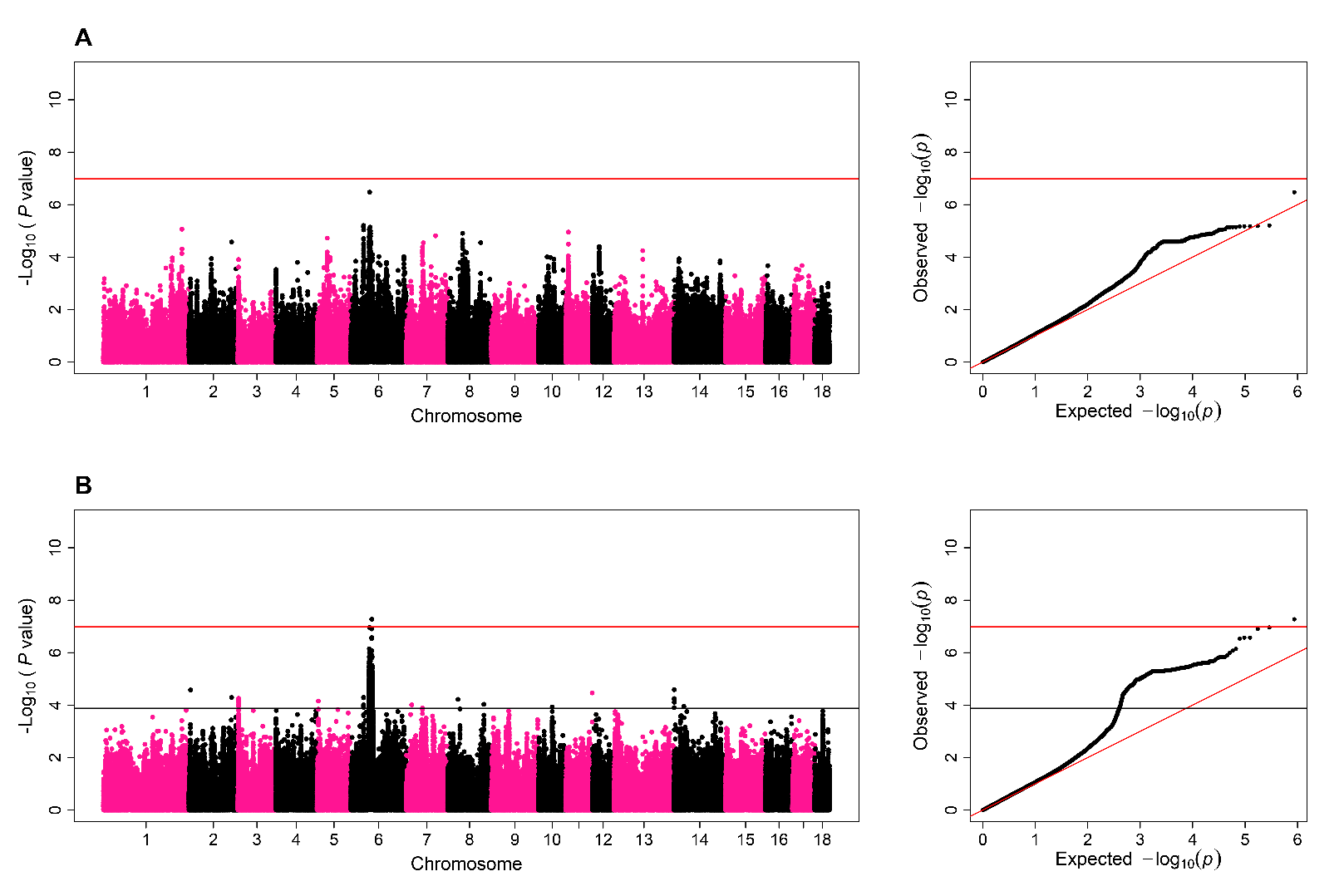
**

**Manhattan plot and QQ-plot for the genome-wide association study of additive (A) and dominance/recessive (B) effects on distal cytoplasmic droplets.** In the Manhattan plots of the GWAS for both additive and dominance/recessive effects, each dot represents a SNP. The *x*-axis shows the chromosome position of SNP and the *y*-axis shows −log_10_(*P*-values) for the association test. The red line shows the threshold for significant associations according to the Bonferroni criterion. The black line shows the threshold for suggestive associations according to a false discovery rate of 5%. In the QQ-plot of the GWAS for both additive and dominance/recessive effects, each dot also represents a SNP but the *x*-axis and *y*-axis show the expected and observed distribution of −log_10_(*P*-values), respectively.

**
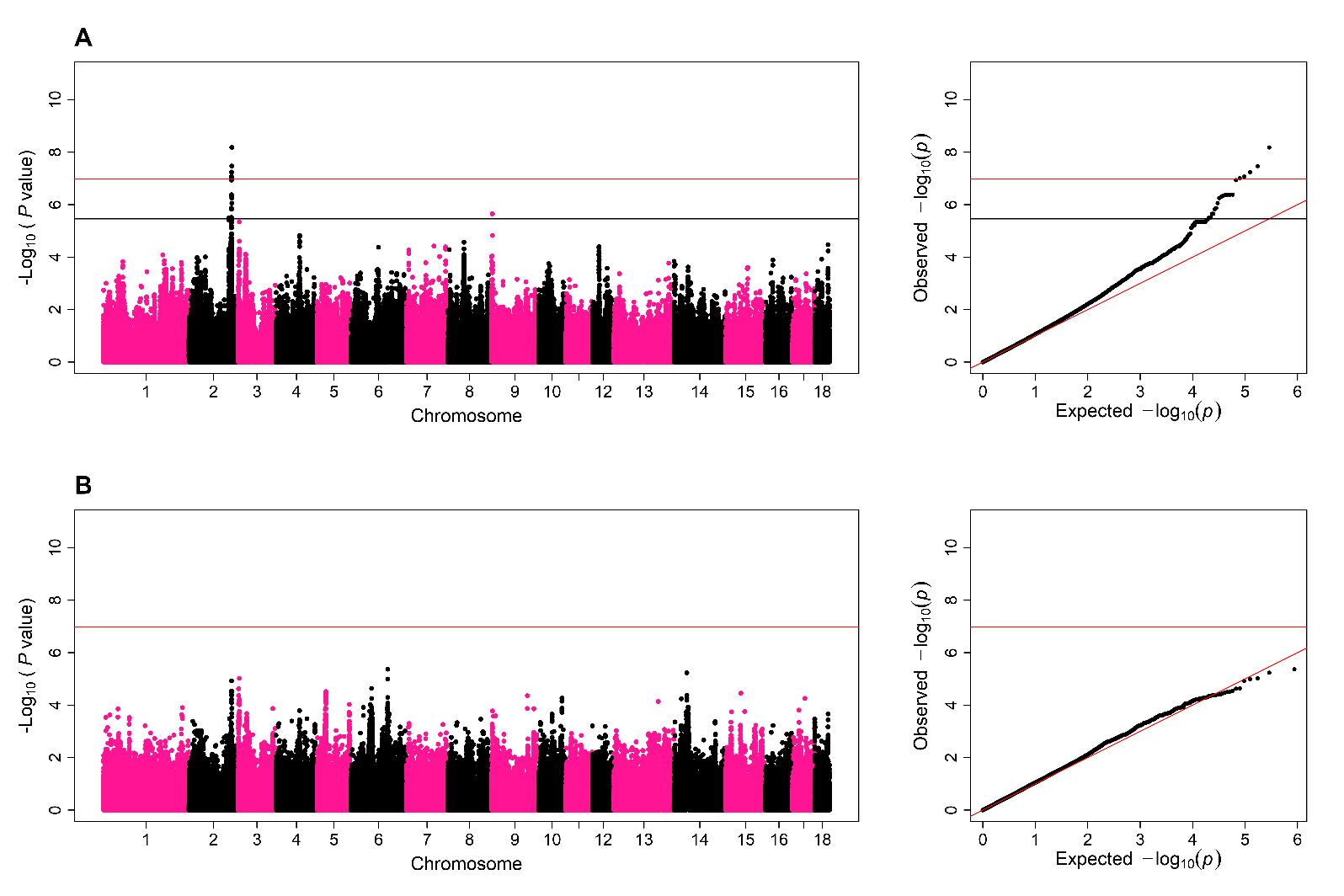
**

**Manhattan plot and QQ-plot for the genome-wide association study of additive (A) and dominance/recessive (B) effects on distal midpiece reflex.** In the Manhattan plots of the GWAS for both additive and dominance/recessive effects, each dot represents a SNP. The *x*-axis shows the chromosome position of SNP and the *y*-axis shows −log_10_(*P*-values) for the association test. The red line shows the threshold for significant associations according to the Bonferroni criterion. The black line shows the threshold for suggestive associations according to a false discovery rate of 5%. In the QQ-plot of the GWAS for both additive and dominance/recessive effects, each dot also represents a SNP but the *x*-axis and *y*-axis show the expected and observed distribution of −log_10_(*P*-values), respectively.

**
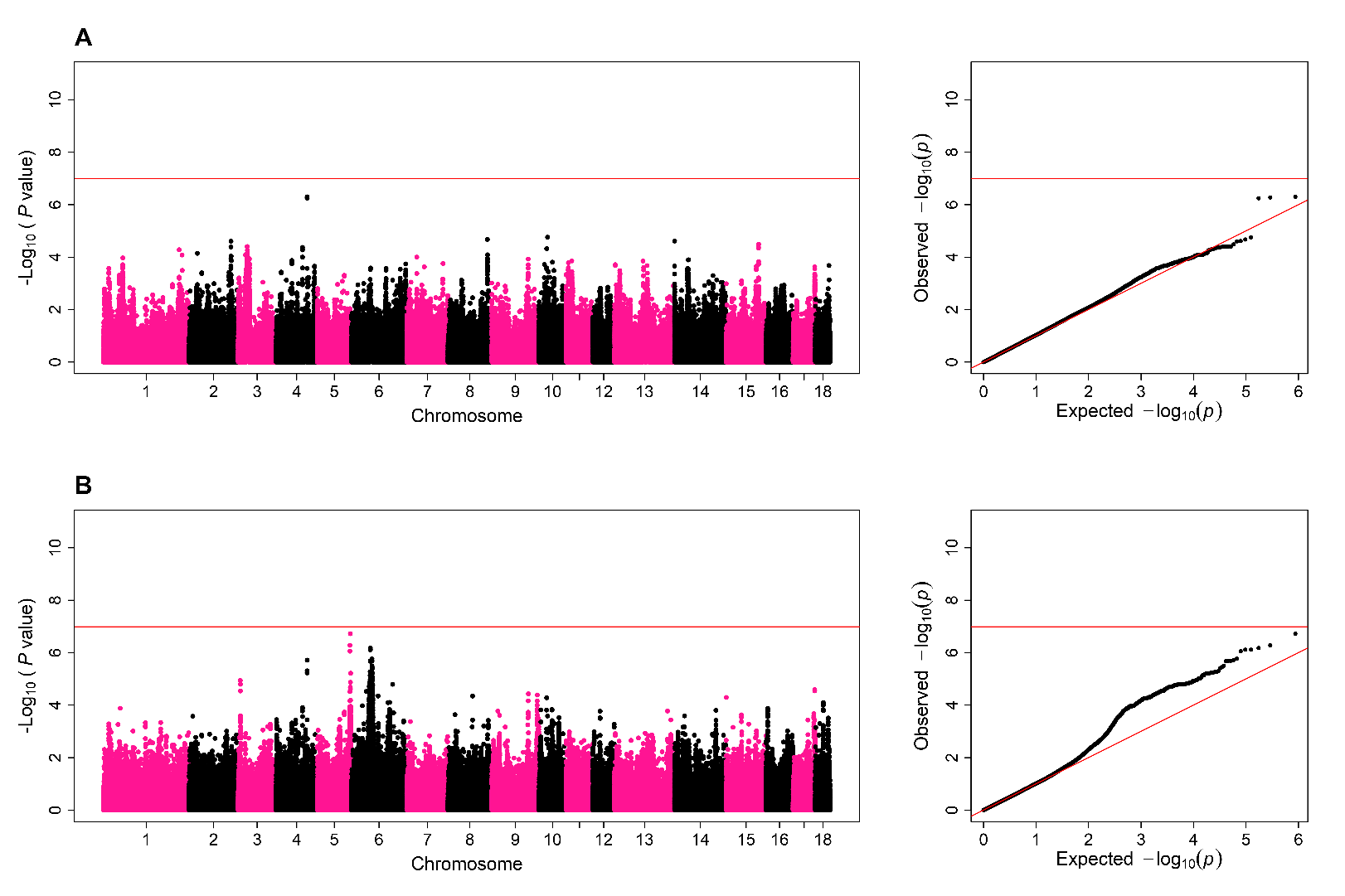
**

**Manhattan plot and QQ-plot for the genome-wide association study of additive (A) and dominance/recessive (B) effects on bent tail.** In the Manhattan plots of the GWAS for both additive and dominance/recessive effects, each dot represents a SNP. The *x*-axis shows the chromosome position of SNP and the *y*-axis shows −log_10_(*P*-values) for the association test. The red line shows the threshold for significant associations according to the Bonferroni criterion. The black line shows the threshold for suggestive associations according to a false discovery rate of 5%. In the QQ-plot of the GWAS for both additive and dominance/recessive effects, each dot also represents a SNP but the *x*-axis and *y*-axis show the expected and observed distribution of −log_10_(*P*-values), respectively.

**
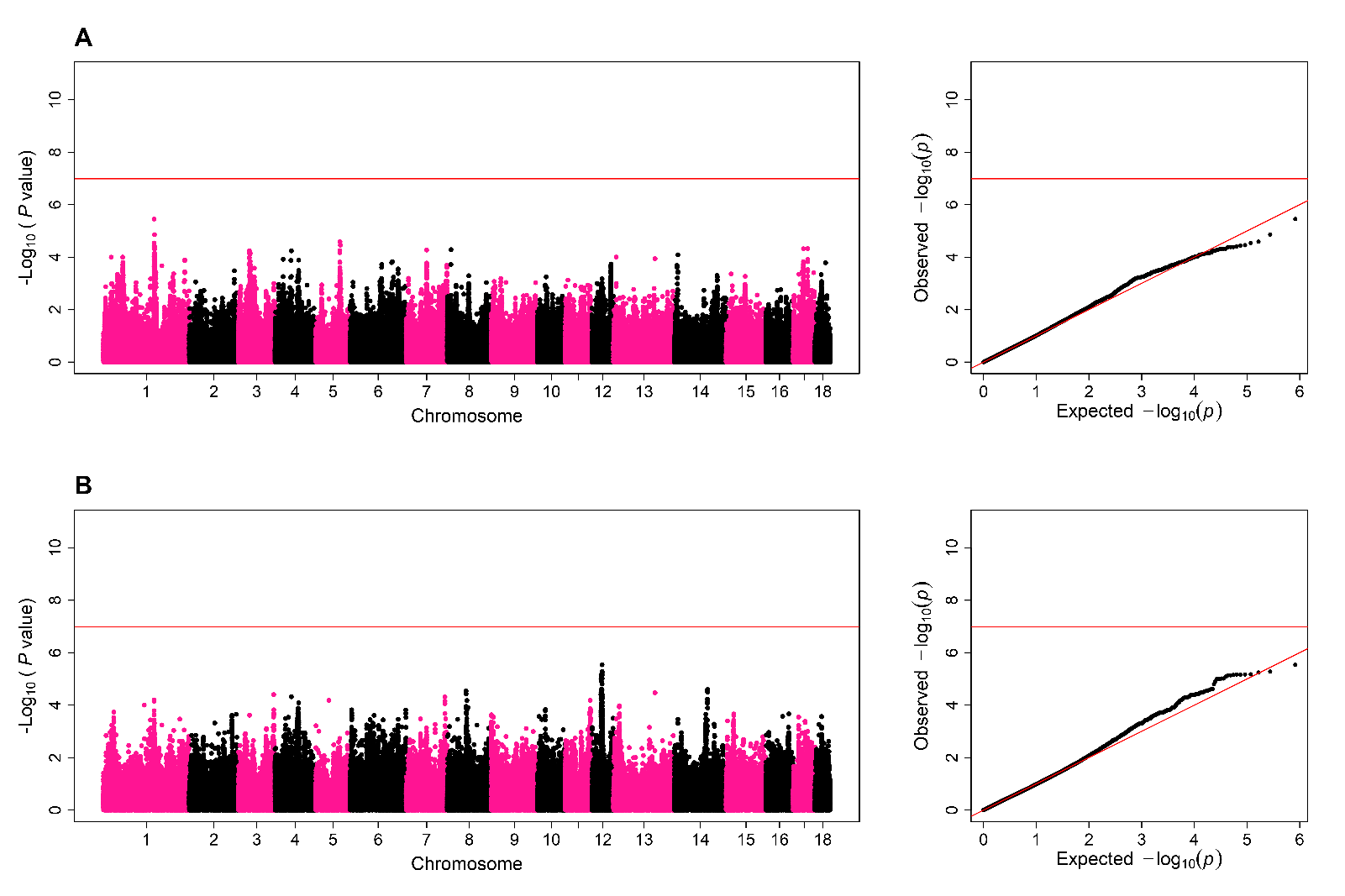
**

**Manhattan plot and QQ-plot for the genome-wide association study of additive (A) and dominance/recessive (B) effects on abnormal acrosome.** In the Manhattan plots of the GWAS for both additive and dominance/recessive effects, each dot represents a SNP. The *x*-axis shows the chromosome position of SNP and the *y*-axis shows −log_10_(*P*-values) for the association test. The red line shows the threshold for significant associations according to the Bonferroni criterion. The black line shows the threshold for suggestive associations according to a false discovery rate of 5%. In the QQ-plot of the GWAS for both additive and dominance/recessive effects, each dot also represents a SNP but the *x*-axis and *y*-axis show the expected and observed distribution of −log_10_(*P*-values), respectively.

**
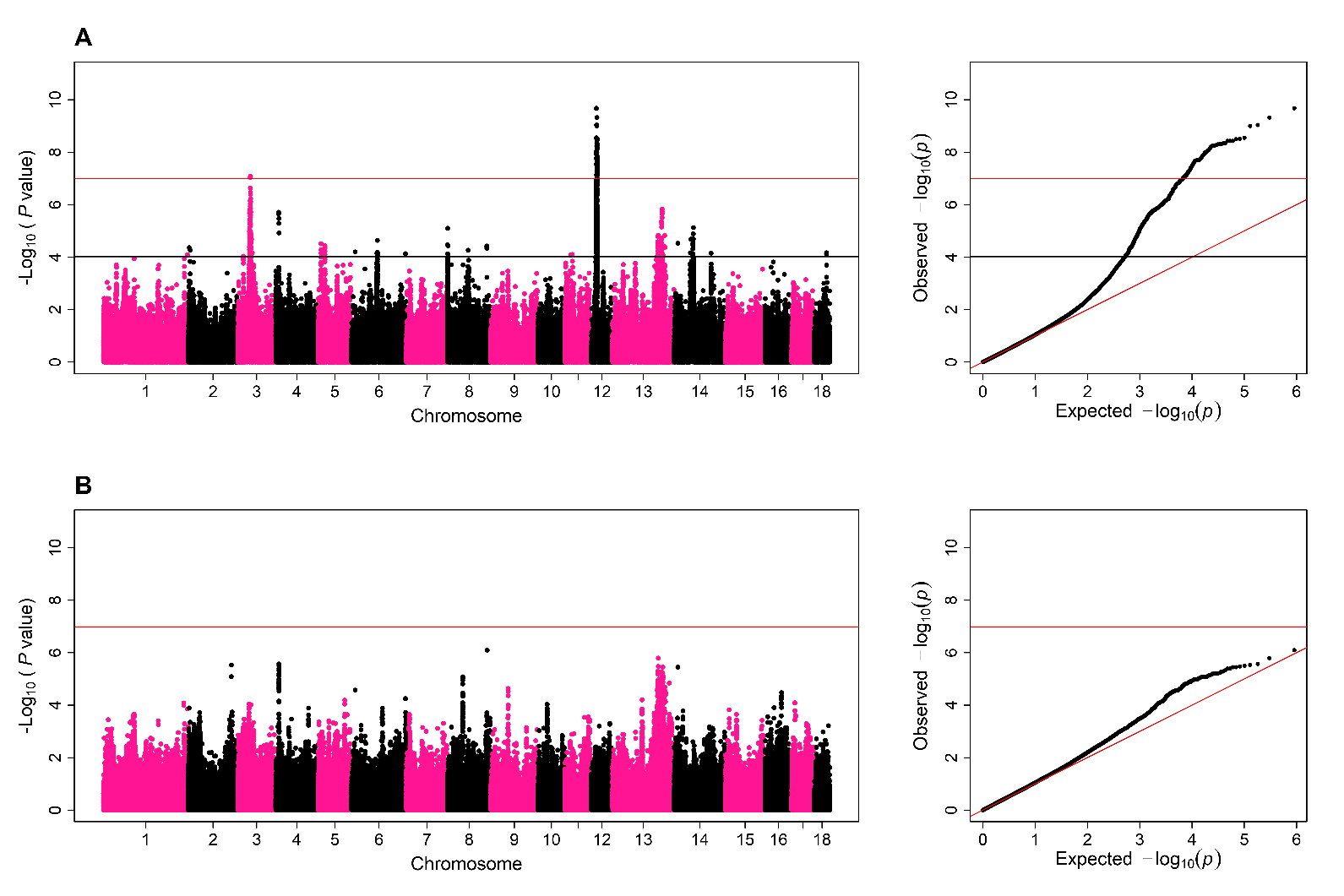
**

**Manhattan plot and QQ-plot for the genome-wide association study of additive (A) and dominance/recessive (B) effects on abnormal head.** In the Manhattan plots of the GWAS for both additive and dominance/recessive effects, each dot represents a SNP. The *x*-axis shows the chromosome position of SNP and the *y*-axis shows −log_10_(*P*-values) for the association test. The red line shows the threshold for significant associations according to the Bonferroni criterion. The black line shows the threshold for suggestive associations according to a false discovery rate of 5%. In the QQ-plot of the GWAS for both additive and dominance/recessive effects, each dot also represents a SNP but the *x*-axis and *y*-axis show the expected and observed distribution of −log_10_(*P*-values), respectively.
