# Supplementary material for: A genome-wide association study reveals additive and recessive alleles affecting male fertility in pigs
Source: J Anim Sci Biotechnol. 2025 Dec 15;16:171. doi: 10.1186/s40104-025-01312-8 (PMC12703936; doi:10.1186/s40104-025-01312-8)
Supplement: Supplementary file 2 — Additional file 2. Summary of QTL significantly associated with semen traits. This table describes the positions of significant QTL, number of significant SNPs and the −log10( (P-value) of lead SNPs. [file 40104_2025_1312_MOESM2_ESM.docx]

**Additional file 2** Summary of QTL significantly associated with semen traits

| **Trait** | **Genomic region** | | | | | | **Additive**  **−log_10_(*P*-value)^b^** | **Dominance/Recessive**  **−log_10_(*P*-value)^b^** |
| --- | --- | --- | --- | --- | --- | --- | --- | --- |
|  | **Chr^a^** | **Start** | **End** | **Size, Mb** | **# SNPs Bonferroni^b^** | **# SNPs**  **FDR 5%^c^** |  |  |
| Ejaculate concentration | SSC3 | 35.3 | 44.2 | 8.8 | 3 | 116 | 7.2 | - |
|  | SSC12 | 6.6 | 9.0 | 2.4 | 4 | 14 | 8.9 | 7.6 |
|  | SSC14 | 104.4 | 106.7 | 2.3 | 3 | 5 | - | 7.8 |
| Number of sperms in ejaculate | SSC3 | 42.5 | 44.5 | 2.0 | 1 | 41 | 7.3 | - |
|  | SSC3 | 35.7 | 37.7 | 2.0 | 1 | 32 | - | 7.1 |
| Proximal cytoplasmic droplets | SSC14 | 45.5 | 62.7 | 17.2 | 38 | 63 | 8.6 | - |
| Distal cytoplasmic droplets | SSC6 | 62.7 | 64.7 | 2.0 | 1 | 177 | - | 7.3 |
| Distal midpiece reflex | SSC2 | 135.2 | 137.9 | 2.7 | 6 | 17 | 12.4 | - |
| Abnormal head | SSC3 | 38.5 | 44.1 | 5.6 | 4 | 82 | 7.1 | - |
|  | SSC12 | 14.9 | 19.6 | 4.7 | 64 | 558 | 9.7 | - |

^a^Chr = Chromosome

^b^The Bonferroni threshold was −log_10_(*P*-value) > 7.0

^c^FDR 5% = False discovery rate of 5%. The thresholds using a false discovery rate of 5% differed between traits and ranged between −log_10_(*P*-value) > 4.0 and −log_10_(*P*-value) > 5.6. Non-significant estimates are not given and indicated with a dash
