# Supplementary material for: A genome-wide association study reveals additive and recessive alleles affecting male fertility in pigs
Source: J Anim Sci Biotechnol. 2025 Dec 15;16:171. doi: 10.1186/s40104-025-01312-8 (PMC12703936; doi:10.1186/s40104-025-01312-8)
Supplement: Supplementary file 3 — Additional file 3. Summary of suggestive QTL associated with semen traits. Description: This table describes the positions of suggestive QTL according to a false discovery rate of 5%, the number of suggestive SNPs and the −log10(P-value) of lead SNPs. [file 40104_2025_1312_MOESM3_ESM.docx]

**Additional file 3** Summary of suggestive QTL associated with semen traits in the pure line

| **Trait** | **Genomic region** | | | | **# SNPs**  **FDR 5%^b^** | **Additive**  **−log_10_(*P*-value)^c^** | **Dominance/**  **Recessive**  **−log_10_(*P*-value)^c^** |
| --- | --- | --- | --- | --- | --- | --- | --- |
|  | **Chr^a^** | **Start** | **End** | **Size, Mb** |  |  |  |
| Volume | SSC1 | 64.4 | 71.5 | 7.1 | 3 | 6.2 | - |
|  | SSC3 | 20.2 | 22.2 | 2.0 | 3 | - | 5.9 |
|  | SSC16 | 30.2 | 33.4 | 3.2 | 72 | 6.1 | - |
| Ejaculate concentration | SSC3 | 27.3 | 29.4 | 2.1 | 3 | 4.7 | - |
|  | SSC3 | 11.4 | 13.4 | 2.0 | 3 | - | 4.7 |
|  | SSC8 | 2.2 | 5.6 | 3.4 | 7 | - | 5.6 |
| Number of sperms in ejaculate | SSC3 | 19.7 | 22.2 | 2.5 | 8 | - | 6.4 |
|  | SSC15 | 116.2 | 118.6 | 2.5 | 5 | 5.3 | - |
| Total motility of fresh semen | SSC6 | 75.1 | 77.2 | 2.1 | 4 | 6.0 | - |
| Progressive motility of fresh semen | SSC6 | 52.2 | 59.5 | 7.4 | 227 | - | 6.1 |
| Total cytoplasmic droplets | SSC2 | 85.4 | 89.4 | 3.9 | 16 | 5.2 | - |
|  | SSC6 | 52.2 | 59.5 | 7.3 | 312 | 6.8 | - |
|  | SSC6 | 52.1 | 67.0 | 14.9 | 977 | - | 4.0 |
|  | SSC6 | 157.6 | 156.5 | 2.2 | 16 | - | 5.5 |
|  | SSC9 | 38.7 | 37.7 | 2.1 | 13 | - | 4.3 |
|  | SSC10 | 42.5 | 41.5 | 2.0 | 5 | 4.6 | - |
|  | SSC11 | 9.5 | 8.2 | 2.7 | 116 | 6.4 | - |
|  | SSC14 | 63.8 | 59.4 | 14.5 | 52 | 5.4 | - |
|  | SSC15 | 4.3 | 3.2 | 2.0 | 4 | - | 4.1 |
| Proximal cytoplasmic droplets | SSC2 | 85.4 | 88.3 | 10.7 | 5 | 5.5 | - |
|  | SSC11 | 7.2 | 9.5 | 2.3 | 11 | 5.2 | - |
| Distal cytoplasmic droplets | SSC3 | 1.6 | 3.9 | 2.3 | 8 | - | 4.3 |
|  | SSC6 | 36.7 | 38.7 | 2.0 | 8 | - | 4.3 |
|  | SSC8 | 119.3 | 121.3 | 2.0 | 3 | - | 4.0 |
|  | SSC14 | 0 | 1.9 | 1.9 | 4 | - | 4.6 |
| Bent tail | SSC4 | 101.2 | 103.4 | 2.2 | 3 | 6.3 | - |
|  | SSC5 | 100.4 | 103.3 | 2.9 | 3 | - | 6.7 |
|  | SSC6 | 55.6 | 65.9 | 10.3 | 10 | - | 6.2 |
| Abnormal head | SSC4 | 7.0 | 10.0 | 3.0 | 5 | 5.7 | - |
|  | SSC4 | 7.9 | 10.1 | 2.1 | 6 | - | 5.6 |
|  | SSC5 | 9.7 | 22.4 | 12.8 | 11 | 4.5 | - |
|  | SSC6 | 77.0 | 80.2 | 3.2 | 9 | 4.6 | - |
|  | SSC8 | 0 | 2.8 | 2.8 | 4 | 5.1 | - |
|  | SSC8 | 129.5 | 132.6 | 3.2 | 7 | 4.4 | - |
|  | SSC13 | 152.8 | 179.3 | 26.5 | 62 | 5.8 | - |
|  | SSC13 | 152.9 | 172.6 | 19.7 | 10 | - | 5.8 |
|  | SSC14 | 43.4 | 54.4 | 11.0 | 21 | 5.1 | - |
|  | SSC18 | 44.0 | 46.0 | 2.0 | 8 | 4.2 | - |

^a^Chr = Chromosome

^b^FDR 5% = False discovery rate of 5%. The thresholds using a false discovery rate of 5% differed between traits and ranged between −log(*P*-value) > 4.0 and −log(*P*-value) > 5.6

^c^The thresholds using a false discovery rate of 5% differed between traits and ranged from −log_10_(*P*-value) > 4.0 to −log_10_(*P*-value) > 5.6. Non-suggestive effects are indicated with a dash
