# Supplementary material for: A genome-wide association study reveals additive and recessive alleles affecting male fertility in pigs
Source: J Anim Sci Biotechnol. 2025 Dec 15;16:171. doi: 10.1186/s40104-025-01312-8 (PMC12703936; doi:10.1186/s40104-025-01312-8)
Supplement: Supplementary file 4 — Additional file 4. Estimates of the minor allele frequency of lead SNPs over time. [file 40104_2025_1312_MOESM4_ESM.docx]

**Additional file 4** Estimates of the minor allele frequency of lead SNPs over time

|  | **Trait** | **Lead SNP** | **Minor allele frequency _(SE)_** | | | | | | | $\boldsymbol{\Delta}_{\boldsymbol{Freq}}$**^a^** |
| --- | --- | --- | --- | --- | --- | --- | --- | --- | --- | --- |
|  |  |  | **2013** | **2014** | **2015** | **2016** | **2017** | **2018** | **2019** |  |
|  | **Concentration** | SSC3: 43.2 | 0.40 _(0.03)_ | 0.40 _(0.02)_ | 0.41 _(0.02)_ | 0.42 _(0.02)_ | 0.51 _(0.02)_ | 0.54 _(0.02)_ | 0.55 _(0.02)_ | 0.02 |
|  |  | SSC12: 7.6 | 0.05 _(0.01)_ | 0.02 _(0.01)_ | 0.02 _(0.01)_ | 0.03 _(0.01)_ | 0.03 _(0.01)_ | 0.02 _(0.01)_ | 0.02 _(0.01)_ | -0.01 |
|  |  | SSC14: 105.4 | 0.11 _(0.02)_ | 0.11 _(0.01)_ | 0.05 _(0.01)_ | 0.07 _(0.01)_ | 0.04 _(0.01)_ | 0.05 _(0.01)_ | 0.04 _(0.01)_ | -0.01 |
|  | **Number of sperm cells** | SSC3: 43.5 | 0.41 _(0.03)_ | 0.42 _(0.02)_ | 0.43 _(0.02)_ | 0.42 _(0.02)_ | 0.50 _(0.02)_ | 0.54 _(0.02)_ | 0.55 _(0.02)_ | 0.02 |
|  |  | SSC3: 36.7 | 0.35 _(0.03)_ | 0.35 _(0.02)_ | 0.37 _(0.02)_ | 0.38 _(0.02)_ | 0.46 _(0.02)_ | 0.51 _(0.02)_ | 0.51 _(0.02)_ | 0.02 |
|  | **Proximal cytoplasmic droplets** | SSC14: 46.5 | 0.13 _(0.02)_ | 0.09 _(0.01)_ | 0.08 _(0.01)_ | 0.06 _(0.01)_ | 0.08 _(0.01)_ | 0.06 _(0.01)_ | 0.10 _(0.01)_ | -0.01 |
|  | **Distal cytoplasmic droplets** | SSC6: 63.7 | 0.35 _(0.03)_ | 0.32 _(0.02)_ | 0.34 _(0.02)_ | 0.35 _(0.02)_ | 0.33 _(0.02)_ | 0.31 _(0.02)_ | 0.32 _(0.02)_ | -0.01 |
|  | **Distal midpiece reflex** | SSC2: 136.6 | 0.34 _(0.03)_ | 0.28 _(0.02)_ | 0.36 _(0.02)_ | 0.41 _(0.02)_ | 0.42 _(0.02)_ | 0.47 _(0.02)_ | 0.50 _(0.02)_ | 0.03 |
|  | **Abnormal head** | SSC3: 42.0 | 0.39 _(0.03)_ | 0.38 _(0.02)_ | 0.41 _(0.02)_ | 0.41 _(0.02)_ | 0.49 _(0.02)_ | 0.52 _(0.02)_ | 0.56 _(0.02)_ | 0.02 |
|  |  | SSC12: 16.1 | 0.48 _(0.03)_ | 0.49 _(0.02)_ | 0.45 _(0.02)_ | 0.40 _(0.02)_ | 0.46 _(0.02)_ | 0.42 _(0.02)_ | 0.41 _(0.02)_ | -0.01 |

^a^$\Delta_{Freq}$ = predicted change in allele frequency of lead SNPs with year of birth of the boars in the population
