# Supplementary material for: A genome-wide association study reveals additive and recessive alleles affecting male fertility in pigs
Source: J Anim Sci Biotechnol. 2025 Dec 15;16:171. doi: 10.1186/s40104-025-01312-8 (PMC12703936; doi:10.1186/s40104-025-01312-8)
Supplement: Supplementary file 5 — Additional file 5. The effect of lead SNPs on all semen traits. [file 40104_2025_1312_MOESM5_ESM.docx]

**Additional file 5** The effect of lead SNPs on all semen traits

| **Lead SNP** | **Trait** | $\boldsymbol{\beta}_{\boldsymbol{AA}}$ | $\boldsymbol{\beta}_{\boldsymbol{Aa}}$ | $\boldsymbol{\beta}_{\boldsymbol{aa}}$ | **Additive**  **−log_10_(*P*-value)^a^** | **Dominance/Recessive**  **−log_10_(*P*-value)^a^** |
| --- | --- | --- | --- | --- | --- | --- |
| SSC2: 136.6 | Volume | - | - | - | - | - |
|  | Concentration | - | - | - | - | - |
|  | Number of sperm cells | - | - | - | - | - |
|  | Total motility of fresh semen | 0.6 _(0.4)_ | 0 | -2.2 _(0.5)_ | 3.5 | - |
|  | Total motility after 3 days of storage | - | - | - | - | - |
|  | Progressive motility of fresh semen | 0.9 _(0.5)_ | 0 | -2.6 _(0.6)_ | 3.5 | - |
|  | Progressive motility after 3 days of storage | - | - | - | - | - |
|  | Total morphological abnormalities | -0.6 _(2.0)_ | 0 | 17.0 _(2.5)_ | 5.4 | 4.1 |
|  | Total cytoplasmatic droplets | - | - | - | - | - |
|  | Proximal cytoplasmatic droplets | - | - | - | - | - |
|  | Distal cytoplasmatic droplets | 0.0 _(0.1)_ | 0 | 0.8 _(0.1)_ | 4.6 | 4.3 |
|  | Distal midpiece reflex | -0.5 _(0.2)_ | 0 | 1.4 _(0.2)_ | 12.4 | 4.4 |
|  | Bent tail | - | - | - | - | - |
|  | Abnormal head | - | - | - | - | - |
|  | Abnormal acrosome | - | - | - | - | - |
| SSC3: 36.7 | Volume | - | - | - | - | - |
|  | Concentration | 1.4 _(1.6)_ | 0 | -14.7 _(1.9)_ | 4.8 | 6.3 |
|  | Number of sperm cells | 0.6 _(0.6)_ | 0 | -5.9 _(0.7)_ | 4.9 | 7.1 |
|  | Total motility of fresh semen | - | - | - | - | - |
|  | Total motility after 3 days of storage | - | - | - | - | - |
|  | Progressive motility of fresh semen | - | - | - | - | - |
|  | Progressive motility after 3 days of storage | - | - | - | - | - |
|  | Total morphological abnormalities | - | - | - | - | - |
|  | Total cytoplasmatic droplets | - | - | - | - | - |
|  | Proximal cytoplasmatic droplets | - | - | - | - | - |
|  | Distal cytoplasmatic droplets | - | - | - | - | - |
|  | Distal midpiece reflex | - | - | - | - | - |
|  | Bent tail | - | - | - | - | - |
|  | Abnormal head | - | - | - | - | - |
|  | Abnormal acrosome | - | - | - | - | - |
| SSC3: 42.0 | Volume | - | - | - | - | - |
|  | Concentration | 2.2 _(1.6)_ | 0 | -13.1 _(1.8)_ | 4.3 | 4.3 |
|  | Number of sperm cells | 1.1 _(0.6)_ | 0 | -4.8 _(0.7)_ | 3.7 | 4.3 |
|  | Total motility of fresh semen | - | - | - | - | - |
|  | Total motility after 3 days of storage | - | - | - | - | - |
|  | Progressive motility of fresh semen | - | - | - | - | - |
|  | Progressive motility after 3 days of storage | - | - | - | - | - |
|  | Total morphological abnormalities | - | - | - | - | - |
|  | Total cytoplasmatic droplets | - | - | - | - | - |
|  | Proximal cytoplasmatic droplets | - | - | - | - | - |
|  | Distal cytoplasmatic droplets | - | - | - | - | - |
|  | Distal midpiece reflex | - | - | - | - | - |
|  | Bent tail | - | - | - | - | - |
|  | Abnormal head | -0.1 _(0.1)_ | 0 | 0.3 _(0.1)_ | 7.1 | - |
|  | Abnormal acrosome | - | - | - | - | - |
| SSC3: 43.2 | Volume | - | - | - | - | - |
|  | Concentration | 4.5 _(1.7)_ | 0 | -12.8 _(1.8)_ | 7.2 | 3.0 |
|  | Number of sperm cells | 1.7 _(0.7)_ | 0 | -4.9 _(0.7)_ | 6.1 | 3.5 |
|  | Total motility of fresh semen | - | - | - | - | - |
|  | Total motility after 3 days of storage | - | - | - | - | - |
|  | Progressive motility of fresh semen | - | - | - | - | - |
|  | Progressive motility after 3 days of storage | - | - | - | - | - |
|  | Total morphological abnormalities | - | - | - | - | - |
|  | Total cytoplasmatic droplets | - | - | - | - | - |
|  | Proximal cytoplasmatic droplets | - | - | - | - | - |
|  | Distal cytoplasmatic droplets | - | - | - | - | - |
|  | Distal midpiece reflex | - | - | - | - | - |
|  | Bent tail | - | - | - | - | - |
|  | Abnormal head | -0.2 _(0.1)_ | 0 | 0.2 _(0.1)_ | 4.8 | - |
|  | Abnormal acrosome | - | - | - | - | - |
| SSC3: 43.5 | Volume | - | - | - | - | - |
|  | Concentration | 3.7 _(1.7)_ | 0 | -12.9 _(1.8)_ | 6.4 | 3.9 |
|  | Number of sperm cells | 1.7 _(0.6)_ | 0 | -5.2 _(0.7)_ | 7.3 | 4.5 |
|  | Total motility of fresh semen | - | - | - | - | - |
|  | Total motility after 3 days of storage | - | - | - | - | - |
|  | Progressive motility of fresh semen | - | - | - | - | - |
|  | Progressive motility after 3 days of storage | - | - | - | - | - |
|  | Total morphological abnormalities | - | - | - | - | - |
|  | Total cytoplasmatic droplets | - | - | - | - | - |
|  | Proximal cytoplasmatic droplets | - | - | - | - | - |
|  | Distal cytoplasmatic droplets | - | - | - | - | - |
|  | Distal midpiece reflex | - | - | - | - | - |
|  | Bent tail | - | - | - | - | - |
|  | Abnormal head | -0.1 _(0.1)_ | 0 | 0.2 _(0.1)_ | 3.6 | - |
|  | Abnormal acrosome | - | - | - | - | - |
| SSC6: 63.7 | Volume | - | - | - | - | - |
|  | Concentration | - | - | - | - | - |
|  | Number of sperm cells | - | - | - | - | - |
|  | Total motility of fresh semen | -0.5 _(0.4)_ | 0 | -2.2 _(0.6)_ | - | 3.9 |
|  | Total motility after 3 days of storage | -0.6 _(0.4)_ | 0 | -2.4 _(0.7)_ | - | 3.8 |
|  | Progressive motility of fresh semen | -0.6 _(0.4)_ | 0 | -2.3 _(0.7)_ | - | 3.8 |
|  | Progressive motility after 3 days of storage | -0.4 _(0.4)_ | 0 | -2.3 _(0.6)_ | - | 4.0 |
|  | Total morphological abnormalities | - | - | - | - | - |
|  | Total cytoplasmatic droplets | -0.5 _(2.0)_ | 0 | 20.2 _(3.2)_ | 3.1 | 7.0 |
|  | Proximal cytoplasmatic droplets | -0.2 _(0.2)_ | 0 | 1.3 _(0.3)_ | - | 4.2 |
|  | Distal cytoplasmatic droplets | 0 _(0.1)_ | 0 | 1.1 _(0.2)_ | - | 7.3 |
|  | Distal midpiece reflex | 0.5 _(0.1)_ | 0 | 0.5 _(0.2)_ | - | 4.3 |
|  | Bent tail | 1.9 _(0.8)_ | 0 | 5.9 _(1.4)_ | - | 5.7 |
|  | Abnormal head | - | - | - | - | - |
|  | Abnormal acrosome | - | - | - | - | - |
| SSC12: 7.6 | Volume | - | - | - | - | - |
|  | Concentration | -0.8 _(3.1)_ | 0 | 80.8 _(15.0)_ | 8.9 | 7.6 |
|  | Number of sperm cells | - | - | - | - | - |
|  | Total motility of fresh semen | - | - | - | - | - |
|  | Total motility after 3 days of storage | - | - | - | - | - |
|  | Progressive motility of fresh semen | - | - | - | - | - |
|  | Progressive motility after 3 days of storage | - | - | - | - | - |
|  | Total morphological abnormalities | - | - | - | - | - |
|  | Total cytoplasmatic droplets | - | - | - | - | - |
|  | Proximal cytoplasmatic droplets | - | - | - | - | - |
|  | Distal cytoplasmatic droplets | - | - | - | - | - |
|  | Distal midpiece reflex | - | - | - | - | - |
|  | Bent tail | - | - | - | - | - |
|  | Abnormal head | - | - | - | - | - |
|  | Abnormal acrosome | - | - | - | - | - |
| SSC12: 16.1 | Volume | - | - | - | - | - |
|  | Concentration | - | - | - | - | - |
|  | Number of sperm cells | - | - | - | - | - |
|  | Total motility of fresh semen | - | - | - | - | - |
|  | Total motility after 3 days of storage | - | - | - | - | - |
|  | Progressive motility of fresh semen | - | - | - | - | - |
|  | Progressive motility after 3 days of storage | - | - | - | - | - |
|  | Total morphological abnormalities | - | - | - | - | - |
|  | Total cytoplasmatic droplets | - | - | - | - | - |
|  | Proximal cytoplasmatic droplets | - | - | - | - | - |
|  | Distal cytoplasmatic droplets | - | - | - | - | - |
|  | Distal midpiece reflex | - | - | - | - | - |
|  | Bent tail | - | - | - | - | - |
|  | Abnormal head | 0.2 _(0.1)_ | 0 | -0.2 _(0.1)_ | 9.7 | - |
|  | Abnormal acrosome | - | - | - | - | - |
| SSC14: 46.5 | Volume | - | - | - | - | - |
|  | Concentration | - | - | - | - | - |
|  | Number of sperm cells | - | - | - | - | - |
|  | Total motility of fresh semen | - | - | - | - | - |
|  | Total motility after 3 days of storage | - | - | - | - | - |
|  | Progressive motility of fresh semen | - | - | - | - | - |
|  | Progressive motility after 3 days of storage | - | - | - | - | - |
|  | Total morphological abnormalities | - | - | - | - | - |
|  | Total cytoplasmatic droplets | -1.4 _(0.3)_ | 0 | 9.0 _(1.6)_ | 6.1 | 3.7 |
|  | Proximal cytoplasmatic droplets | -0.9 _(0.2)_ | 0 | 6.3 _(0.6)_ | 8.6 | 5.1 |
|  | Distal cytoplasmatic droplets | -0.4 _(0.2)_ | 0 | 3.1 _(0.8)_ | 3.4 | - |
|  | Distal midpiece reflex | - | - | - | - | - |
|  | Bent tail | - | - | - | - | - |
|  | Abnormal head | - | - | - | - | - |
|  | Abnormal acrosome | - | - | - | - | - |
| SSC14: 105.4 | Volume | - | - | - | - | - |
|  | Concentration | 3.3 _(2.2)_ | 0 | 68.4 _(13.9)_ | 6.2 | 7.8 |
|  | Number of sperm cells | 1.6 _(0.9)_ | 0 | 17.9 _(5.4)_ | - | 3.9 |
|  | Total motility of fresh semen | - | - | - | - | - |
|  | Total motility after 3 days of storage | - | - | - | - | - |
|  | Progressive motility of fresh semen | - | - | - | - | - |
|  | Progressive motility after 3 days of storage | - | - | - | - | - |
|  | Total morphological abnormalities | - | - | - | - | - |
|  | Total cytoplasmatic droplets | - | - | - | - | - |
|  | Proximal cytoplasmatic droplets | - | - | - | - | - |
|  | Distal cytoplasmatic droplets | - | - | - | - | - |
|  | Distal midpiece reflex | - | - | - | - | - |
|  | Bent tail | - | - | - | - | - |
|  | Abnormal head | - | - | - | - | - |
|  | Abnormal acrosome | - | - | - | - | - |

^a^Significant effects on multiple traits were considered when −log_10_(*P*-value) > 3.0

The additive and dominance/recessive GWASes *P*-values were log-transformed. The effect of each genotype class (β) was estimated as a class effect in a post-GWAS analysis. AA = homozygotes for the major allele, Aa = heterozygotes and aa = homozygotes for the minor allele. The effect of genotype classes of a lead SNPs were estimated on untransformed traits. Non-significant estimates are indicated with a dash
